# Prolonged Exposure of Primary Human Muscle Cells to Plasma Fatty Acids Associated with Obese Phenotype Induces Persistent Suppression of Muscle Mitochondrial ATP Synthase β Subunit

**DOI:** 10.1371/journal.pone.0160057

**Published:** 2016-08-17

**Authors:** Lee Tran, Paul D. Hanavan, Latoya E. Campbell, Elena De Filippis, Douglas F. Lake, Dawn K. Coletta, Lori R. Roust, Lawrence J. Mandarino, Chad C. Carroll, Christos S. Katsanos

**Affiliations:** 1 Center for Metabolic and Vascular Biology, Arizona State University and Mayo Clinic in Arizona, Scottsdale, Arizona, United States of America; 2 College of Health Solutions, Arizona State University, Phoenix, Arizona, United States of America; 3 School of Life Sciences, Arizona State University, Tempe, Arizona, United States of America; 4 Department of Medicine, Mayo Clinic, Scottsdale, Arizona, United States of America; 5 Department of Physiology, Midwestern University, Glendale, Arizona, United States of America; University of Barcelona, Faculty of Biology, SPAIN

## Abstract

Our previous studies show reduced abundance of the β-subunit of mitochondrial H+-ATP synthase (β-F1-ATPase) in skeletal muscle of obese individuals. The β-F1-ATPase forms the catalytic core of the ATP synthase, and it is critical for ATP production in muscle. The mechanism(s) impairing β-F1-ATPase metabolism in obesity, however, are not completely understood. First, we studied total muscle protein synthesis and the translation efficiency of β-F1-ATPase in obese (BMI, 36±1 kg/m^2^) and lean (BMI, 22±1 kg/m^2^) subjects. Both total protein synthesis (0.044±0.006 vs 0.066±0.006%·h^-1^) and translation efficiency of β-F1-ATPase (0.0031±0.0007 vs 0.0073±0.0004) were lower in muscle from the obese subjects when compared to the lean controls (*P*<0.05). We then evaluated these same responses in a primary cell culture model, and tested the specific hypothesis that circulating non-esterified fatty acids (NEFA) in obesity play a role in the responses observed in humans. The findings on total protein synthesis and translation efficiency of β-F1-ATPase in primary myotubes cultured from a lean subject, and after exposure to NEFA extracted from serum of an obese subject, were similar to those obtained in humans. Among candidate microRNAs (i.e., non-coding RNAs regulating gene expression), we identified miR-127-5p in preventing the production of β-F1-ATPase. Muscle expression of miR-127-5p negatively correlated with β-F1-ATPase protein translation efficiency in humans (r = – 0.6744; *P*<0.01), and could be modeled *in vitro* by prolonged exposure of primary myotubes derived from the lean subject to NEFA extracted from the obese subject. On the other hand, locked nucleic acid inhibitor synthesized to target miR-127-5p significantly increased β-F1-ATPase translation efficiency in myotubes (0.6±0.1 vs 1.3±0.3, in control vs exposure to 50 nM inhibitor; *P*<0.05). Our experiments implicate circulating NEFA in obesity in suppressing muscle protein metabolism, and establish impaired β-F1-ATPase translation as an important consequence of obesity.

## Introduction

The increasing prevalence of obesity is a major health concern due to its associated physiological and metabolic consequences. Obesity, whose cardinal characteristic is excess adiposity, is associated with increase in various health risk factors linked to cardiovascular disease and type 2 diabetes [1]. Fatty acids in obesity originating from either plasma or muscle triacylglycerol deposits are implicated in impaired glucose metabolism in skeletal muscle [2]. In addition to its effects on the cardiovascular system and impaired glucose metabolism, obesity also perturbs muscle protein synthesis [3,4]. The precise mechanism(s) reducing protein synthesis in muscle in obesity are not completely understood. Factors associated with obesity such as insulin resistance, inflammation, and oxidative stress may contribute to impaired muscle protein metabolism in the post-absorptive state [5]. Also, it is possible that fatty acids, either directly or indirectly via their effects on inducing insulin resistance, may reduce protein synthesis in muscle in obesity. We have shown that acute increase in plasma non-esterified fatty acid (NEFA) availability induced by the intravenous infusion of a fat emulsion, although it induced insulin resistance, did not impair the muscle anabolic response to amino acids [6], which suggests disassociation between NEFA-mediated insulin resistance and muscle protein metabolism. However, elevation of NEFA in the basal state impairs muscle protein synthesis [7], indicating a role of plasma NEFA in decreasing muscle protein metabolism in the post-absorptive state. Also, in an animal model of dietary fat-induced obesity, reduced muscle protein synthesis is observed concomitant with increased lipid infiltration in muscle [8]. Taken together, the latter evidence suggests that muscle lipid exposure, which can be either through plasma NEFA or via the action of lipoprotein lipase in circulating triacylglycerols, or triacylglycerol-associated fatty acids from adipocytes interspersed between muscle fibers, can impair muscle protein metabolism in the setting of obesity.

Obesity is associated with reduced abundance of mitochondrial proteins, including the H+-ATP synthase complex [9] involved in muscle ATP production. In line with such evidence, muscle ATP metabolism is impaired in obesity/insulin resistance [10,11], and the expression specifically of the beta subunit of mitochondrial H+-ATP synthase (β-F1-ATPase), which catalyzes the conversion of ADP to ATP, is reduced in the muscle from obese individuals [12]. In liver tissue, reduced β-F1-ATPase has been linked to abnormal ATP content in animal models [13]. Therefore, understanding the metabolism of β-F1-ATPase has important physiological implications in explaining energy homeostasis in muscle in obesity. Although protein abundance is determined by multiple factors, mRNA translation has a primary role in controlling protein expression in skeletal muscle [14], including that of β-F1-ATPase [15,16]. Translation of mRNA can be regulated by epigenetic mechanisms, i.e. DNA methylation, histone modifications, and microRNAs (miRNAs), which allow for long-term adaptations to distinct environmental and physiological challenges.

The β-F1-ATPase is a nuclear encoded enzyme (*ATP5B*), and the synthesis of β-F1-ATPase is known to be subject to regulation by 3’UTR binding factors [17–19]. miRNAs can bind to the 3’UTR of mRNA transcripts and silence translation through several mechanisms such as targeting degradation of the mRNA or blocking translation [20–22]. The latter has been reported for the β-F1-ATPase during cellular development and tumorigenesis [19,23]. More specifically, miR-101-3p and miR-127-5p target the β-F1-ATPase mRNA and inhibit translation without affecting mRNA content. The role of miRNAs in controlling β-F1-ATPase production has not been evaluated in human skeletal muscle or linked to obesity.

The goal of the current study was to investigate putative mechanisms impairing muscle protein, and specifically β-F1-ATPase, metabolism in obesity. We hypothesized that the plasma NEFA profile associated with obesity reduces total protein synthesis in muscle, and at the single protein level suppresses the translation efficiency of β-F1-ATPase, and that the latter effect is mediated by miRNAs. Our investigation was divided into three experimental series: i) determine the effects of obesity on skeletal muscle total protein synthesis and the translation efficiency of β-F1-ATPase, ii) quantify effects on protein metabolism after exposing primary culture myotubes from a lean subject to NEFA extracted from serum of an obese subject, and iii) explore miRNAs as putative intermediates regulating muscle β-F1-ATPase translation.

## Materials and Methods

### Human subjects

The protocol and consent form were approved by the Institutional Review Board (IRB) at Mayo Clinic (IRB #12–004000). All participants provided written informed consent, following a consent procedure approved by the IRB. Healthy, sedentary lean (n = 6, BMI<25) and obese (n = 7, BMI>30) subjects were studied at the Clinical Studies Infusion Unit (CSIU) in Mayo Clinic, Scottsdale, AZ, after screening that included physical examination, medical history, and routine blood and urine analyses. Prior to their physical screening visit, subjects were screened over the phone about medications and supplements that might affect protein, glucose, or fat metabolism. None of the subjects included in the study were taking any medications or supplements. Fasting glucose (YSI analyzer, Yellow Springs, OH) and insulin (ELISA kit, ALPCO Diagnostics, Windham, NH) levels were used to calculate a HOMA-IR [24]. Plasma amino acid concentrations were quantified by high performance liquid chromatography (HPLC) [25] and NEFA by HPLC-mass spectrometry (HPLC-MS) [26]. Body fat percentage (BF %) and fat free mass (FFM) were determined using bioimpedance analysis (BIA 310e, Biodynamics Corp., Shoreline, WA). Subject characteristics are shown in Table 1. Dietary intake (three-day diet record; Table 2) was analyzed using The Food Processor Nutrition Analysis Software (ESHA Research, Salem, OR). Serum NEFA concentrations of the study participants are shown in Table 3.

**Table 1:** Subject characteristics.

	Lean	Obese
Gender (M/F)	3M / 3F	4M / 3F
Age (years)	33 ± 5	39 ± 4
BMI (kg/m^2^)	22 ± 1	36 ± 1‡
Height (cm)	171 ± 5	171 ± 3
Weight (kg)	65 ± 6	108 ± 7‡
Body Fat (%)	22 ± 3	36 ± 2†
Lean Body Mass (kg)	52 ± 6	69 ± 4*
HDL Cholesterol (mM)	1.8 ± 0.2	1.0 ± 0.1‡
Total Cholesterol (mM)	4.1 ± 0.3	4.9 ± 0.3*
Triacylglycerols (mM)	0.6 ± 0.1	2.6 ± 0.6†
Glucose (mM)	4.9 ± 0.2	5.4 ± 0.3
Insulin (pM)	29 ± 3	101 ± 14‡
HOMA-IR	0.9 ± 0.1	3.2 ± 0.7†
HbA1c (%)	5.5 ± 0.1	5.7 ± 0.1

Data are means ± SE

* *P*<0.05

† *P*<0.01

‡ *P*<0.001 vs lean control subjects

**Table 2:** Dietary analysis.

	Lean (n = 6)	Obese (n = 6)
Protein (%)	16.5 ± 1.3	14.7 ± 1.3
Carbohydrates (%)	47.2 ± 4.0	44.2 ± 4.5
Fat (%)	35.5 ± 3.5	41.2 ± 4.8
Saturated Fat (%)	10.1 ± 1.4	13.9 ± 1.6*
Monosaturated Fat (%)	9.0 ± 1.6	6.3 ± 1.9
Polyunsaturated Fat (%)	6.6 ± 1.2	3.2 ± 1.2*
Trans Fatty Acid (%)	0.7 ± 0.2	0.6 ± 0.2
Other Fat (%)	8.2 ± 1.4	17.2 ± 4.7*
Gram Weight (g)	2909 ± 312	2772 ± 201
Calories (kcal)	3240 ± 343	3455 ± 661
Calories from Fat (kcal)	1236 ± 170	1487 ± 421
Calories from Sat Fat (kcal)	332 ± 76	474 ± 101
Protein (g)	122 ± 16	120 ± 13
Carbohydrates (g)	368 ± 55	392 ± 81
Dietary Fiber (g)	27.6 ± 2.7	21.8 ± 3.9
Soluble Fiber (g)	1.9 ± 0.4	0.6 ± 0.3†
Total Sugars (g)	116 ± 23	122 ± 27
Monosaccharides (g)	18.5 ± 2.9	6.4 ± 1.3†
Disaccharides (g)	38.7 ± 20.3	20.8 ± 5.2
Other Carbohydrates (g)	176 ± 30	170 ± 50
Fat (g)	123 ± 24	165 ± 47
Saturated Fat (g)	36.9 ± 8.5	52.7 ± 11.2
Monosaturated Fat (g)	33.9 ± 9.7	18.6 ± 3.5
Polyunsaturated Fat (g)	23.7 ± 6.0	8.9 ± 2.5*
Trans Fatty Acid (g)	2.5 ± 0.9	1.7 ± 0.4
Cholesterol (mg)	518 ± 120	393 ± 55
Water (g)	1466 ± 309	1507 ± 138
Omega-3 Fatty Acid (g)	1.7 ± 0.5	1.1 ± 0.3
Omega-6 Fatty Acid (g)	19.3 ± 5.6	7.4 ± 2.3*

Data are means ± SE

* *P*<0.05

† *P*<0.01 vs lean control subjects.

**Table 3:** Circulating non-esterified fatty acids (uM).

		Lean (n = 6)	Obese (n = 7)
Myristic	14:0	5.6 ± 0.6	6.6 ± 0.6
Palmitic	16:0	143 ± 11	157 ± 10
Palmitoleic	16:1 cis	10.3 ± 1.5	10.1 ± 1.0
Stearic	18:0	72.3 ± 5.3	79.2 ± 5.7
Oleic	18:1 cis	198 ± 21	185 ± 12
Elaidic	18:1 trans	5.5 ± 0.5	7.9 ± 0.9*
Linoleic	18:2 (n-6)	109 ± 6	88 ± 8*
Linolenic	18:3 (n-3)	9.2 ± 1.3	7.5 ± 0.9
Arachidonic	20:4 (n-6)	4.0 ± 0.3	3.5 ± 0.6
PUFA		122 ± 7	99 ± 9*
MUFA		214 ± 22	203 ± 13
SFA		221 ± 10	243 ± 13
PUFA/SFA		0.55 ± 0.02	0.41 ± 0.04†
Total		557 ± 37	545 ± 29

Data are means ± SE

* *P*<0.05

† *P*<0.01, vs lean control subjects

MUFA, monounsaturated fatty acids; PUFA, polyunsaturated fatty acids; SFA, saturated fatty acids.

### Infusion protocol and tissue collection

Details of the protocol used to measure total muscle protein synthesis are described previously [6,27]. Briefly, subjects arrived at the CSIU the morning of the study after a 10-hr overnight fast. An intravenous line was inserted into an antecubital vein for infusions and another one in a retrograde fashion into a dorsal metacarpal vein in the contralateral hand for arterialized blood sampling. Patency was maintained by slow infusion of normal saline. A primed (2.7 umol·kg^-1^) continuous (0.05 umol·kg^-1^·min^-1^) infusion of L-[ring-^13^C_6_]phenylalanine ([^13^C_6_]phe; Cambridge Isotope Laboratories, Inc., Andover, MA) tracer was administered for five hours. Blood samples were collected before the initiation of any infusions, and every fifteen minutes starting at 1.5 hours after the initiation of the tracer infusion. Muscle biopsies were collected at two and five hours after the initiation of the tracer. Percutaneous muscle biopsies (~80 mg) were collected from the *vastus lateralis* muscle using a Bergström cannula. Blood and muscle samples were processed as previously described [28], and enrichments of [^13^C_6_]phe in blood and protein-bound amino acids in muscle were determined using LC-MS/MS [29]. Enrichment was expressed as molar percent excess. The fractional synthesis rate (FSR; %·h^-1^) of total muscle protein was calculated as described in the S1 Appendix.

### Primary tissue culture and non-esterified fatty acid extraction

Tissue culture conditions, reagent sources, and procedures have been previously described [30,31]. The experimental procedures used for tissue culture together with the NEFA extraction used in the tissue culture experiments are described in detail in the S1 Appendix. Differentiated myotubes were exposed to NEFA for a period of one week. As opposed to short term (i.e., hours) culture of differentiated myotubes with lipids [32], typical in cell culture experiments, exposure of the differentiated myotubes to NEFA for a week represents a more physiological *in vivo* circumstance, and where muscle cells are exposed to lower lipid concentrations but for a rather longer period of time.

### Quantification of mRNA/miRNA and protein

For the extraction and quantification of mRNA we used procedures we have previously described [33–35]. Proteins were simultaneously extracted from QIAzol lysates following a protocol adapted from Simões et al. [36]. Details of both the mRNA/miRNA and protein quantification are described in the S1 Appendix.

### Concurrent glucose uptake and protein synthesis

Simultaneous glucose uptake and protein synthesis in the cell culture experiments were determined using the fluorescent glucose analogue 2-deoxy-2-[(7-nitro-2,1,3-benzoxadiazol-4-yl) amino]-D-glucose) and puromycin analogue O-propargyl-puromycin, respectively. Details are provided in the S1 Appendix.

### Inhibition of miR-127-5p experiments and exosome isolation

Details of the experiments for the inhibition of miR-127-5p, as well as the procedures used for the isolation of exosomes in serum are provided in the S1 Appendix.

### DNA methylation

DNA methylation was assessed using the sodium bisulfite sequencing method following procedures we have previously described [33], and the exact details are described in the S1 Appendix.

### Statistical analyses

Data reported are mean±standard error (SE) and significance was determined at the *P*≤0.05 level. Statistical significance was calculated using a one-way analysis of variance (ANOVA) with Tukey’s posthoc test for multiple comparisons, or an unpaired Student’s t-test for two-population comparison. A two-way repeated measures-ANOVA (RM-ANOVA) with Bonferroni correction for multiple comparisons was used for all two-factor analyses. Correlation was determined by calculating a Pearson product-moment correlation coefficient (r). Equality of sample variance and normality were determined using an F-test and Shapiro-Wilk test, respectively. Statistical calculations were made using Graphpad Prism Software (ver 6.0b; La Jolla, CA).

## Results

### Skeletal muscle protein synthesis, dietary analysis and circulating NEFA profile

**Fig 1:**
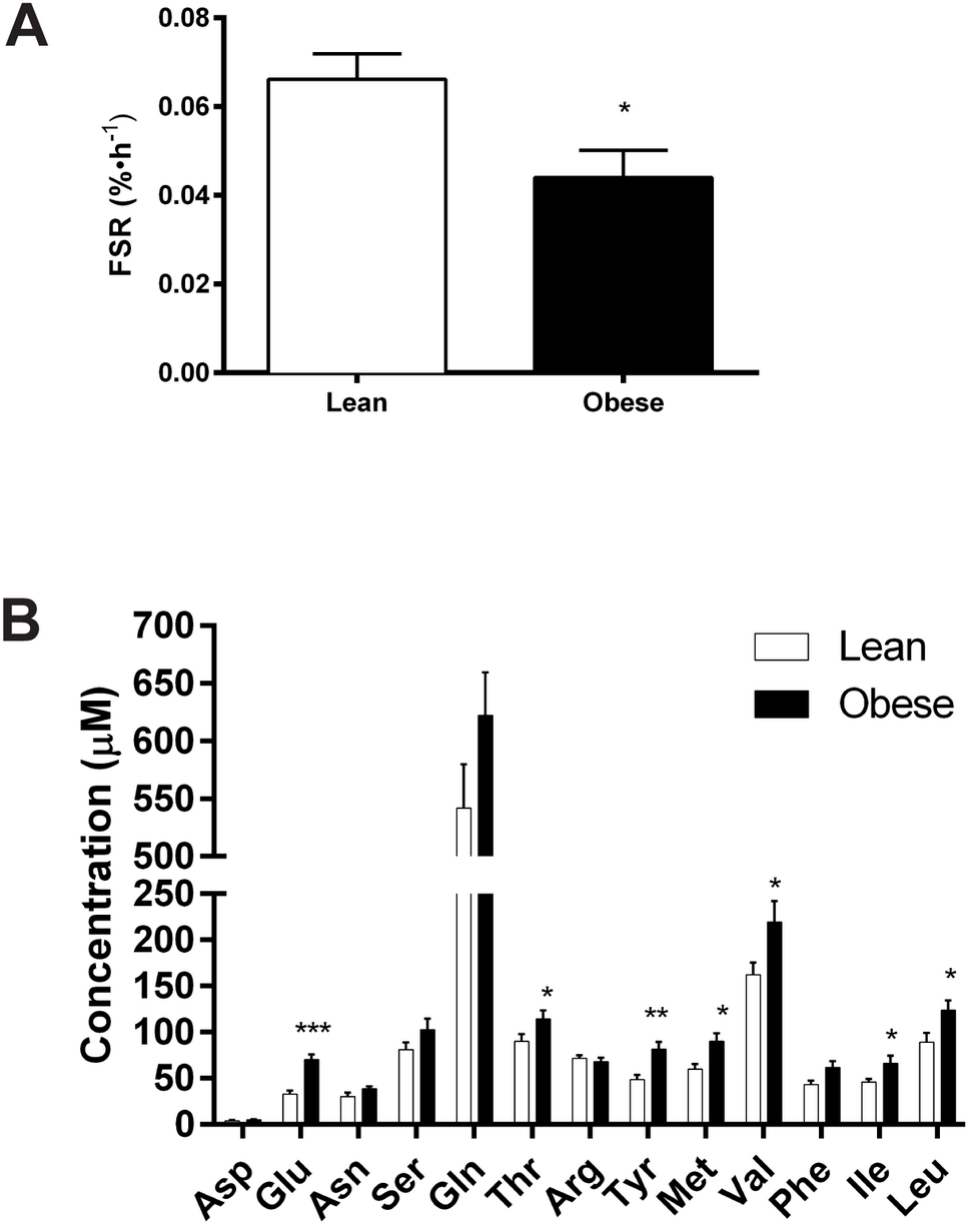
Decreased total protein synthesis in muscle and increased amino acid concentrations in plasma in obese subjects. (A) Fractional synthesis rate (FSR) for total muscle protein in the lean (BMI<25) and obese (BMI>30) subjects. (B) Fasting plasma amino acid concentrations. Data shown are means ±SE, and significance was *P<0.05, **P<0.01, and ***P<0.001 by Student’s unpaired t-test.

We found reduced rate of total protein synthesis in skeletal muscle in obese subjects compared to lean controls (t_(11)_ = 2.608; *P* = 0.012; Fig 1A). We also measured the concentration of plasma amino acids, whose availability regulates muscle protein synthesis. Analysis of the plasma amino acid concentrations revealed an effect of obesity, and many of the measured amino acids were significantly elevated in the obese individuals compared to lean controls (Fig 1B).

Although caloric intake was not different between lean and obese subjects, we found trends in the types of dietary fats consumed (Table 2). Differences in the percent contribution of polyunsaturated and saturated fats in the diet were also reflected in the circulating NEFA of the two subject populations. For example, the ratio of polyunsaturated fatty acids (PUFA) to saturated fatty acids (SFA) (i.e., PUFA/SFA) was significantly lower in the obese subjects (Table 3). We hypothesized that the specific circulating NEFA profile in our obese subjects suppresses muscle protein metabolism. Thus, we explored whether the NEFA profile of our obese subjects suppresses protein metabolism in primary muscle cells from a lean subject and when compared to the NEFA profile typical of the lean phenotype. Extracted serum NEFA from representative subjects from our pool of lean and obese subjects studied during the *in vivo* determination of total muscle protein synthesis were used to represent obese (i.e., Obese NEFA) and lean (i.e., Lean NEFA) NEFA profiles. The representative obese and lean subjects whose NEFA were used in the cell culture experiments (Table 4; right), were selected on the basis of how well their circulating NEFA profiles reflected the average circulating NEFA profiles of our obese and lean subjects, respectively, and as shown in Table 4 (left). Because generally PUFA (i.e., omega-3 fatty acids [37]), or arachidonic acid [38,39] have been linked to favorable effects on muscle protein metabolism, NEFA were also extracted from the serum of a lean subject with relatively higher PUFA concentrations and PUFA/SFA ratio among the lean subjects (i.e., Lean NEFA_(P)_). These NEFA were used to evaluate the specific effects of increased contribution of PUFA to circulating NEFA on muscle protein metabolism.

**Table 4:** Circulating non-esterified fatty acids in the lean and obese subjects (left two columns), and in three subjects whose non-esterified fatty acids extracted for tissue culture experiments (right three columns).

	Lean	Obese	VEH	Lean NEFA	Lean NEFA_(P)_	Obese NEFA
Palmitic (%)	25.7 ± 0.7	28.8 ± 0.6†	21.3	27.3	26.3	29.9
Oleic (%)	35.1 ± 1.7	33.8 ± 0.7	31.8	36.7	38.2	31.7
Elaidic (%)	1.0 ± 0.1	1.4 ± 0.2*	1.7	1.2	0.9	1.6
Linoleic (%)	19.6 ± 0.5	16.1 ± 1.1†	20.9	18.7	18.9	18.7
MUFA (uM)	214 ± 22	203 ± 13	127	244	281	213
PUFA (um)	122 ± 7	99 ± 9*	82	128	144	126
SFA (uM)	221 ± 10	243 ± 13	150	241	250	263
PUFA/SFA	0.55 ± 0.02	0.41 ± 0.04†	0.55	0.53	0.58	0.48
Total (uM)	557 ± 37	545 ± 29	359	613	675	602

Data are means ± SE for the Lean and Obese

* *P*<0.05

† *P*<0.01 vs lean control subjects.

VEH, vehicle, NEFA, non-esterified fatty acids; Lean NEFA, NEFA from a lean subject; Lean NEFA_(P)_, NEFA from a lean subject with higher content of PUFA; Obese NEFA, NEFA from an obese subject; MUFA, monounsaturated fatty acids (sum of palmitoleic acid, oleic acid, and elaidic acid); PUFA, polyunsaturated fatty acids (sum of linoleic acid, α-linolenic acid, and arachidonic acid); SFA, saturated fatty acids (sum of myristic acid, palmitic acid, and stearic acid).

### Exposure of primary human myotubes to NEFA

Differentiated myotubes propagated from a muscle biopsy sample that was obtained from a healthy lean subject were treated with three separate NEFA-containing media, and following the experimental protocol shown in S1A Fig. NEFA extracted from the collected serum of a single, representative lean subject (i.e., Lean NEFA), from a single lean subject with relatively higher PUFA/SFA ratio (i.e., Lean NEFA_(P)_), and a single, representative obese subject (i.e., Obese NEFA). The plasma NEFA profiles associated with the three subjects are shown in Table 4 (right). We observed no qualitative differences in myotube viability or morphology across the cell culture experiments associated with the three NEFA treatments (S1B Fig).

**Fig 2:**
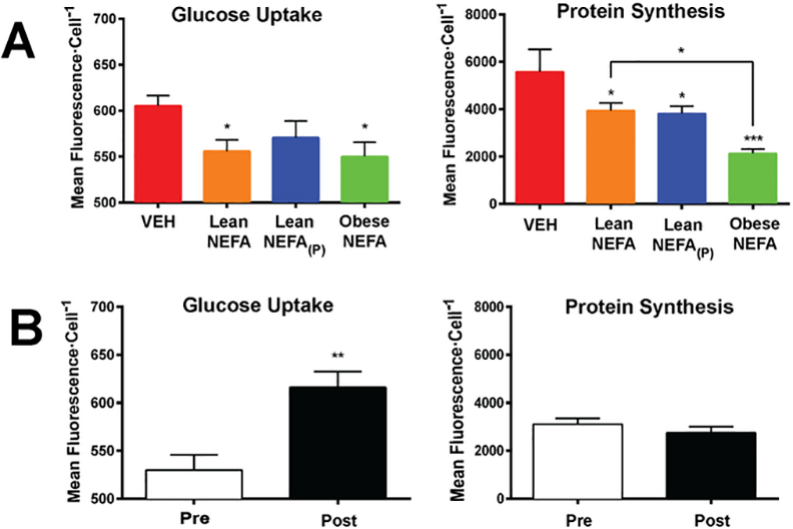
Concurrent assessment of glucose uptake and nascent protein synthesis in primary human myotubes reveals independent regulatory mechanisms. (A) Average responses for glucose uptake and protein synthesis. (B) Corresponding recovery experiments for myotubes exposed to Obese NEFA (Pre) and then incubated for an additional five days in normal growth media (Post). Data shown are means ±SE, n = 6 replicates/group, 10^−3^ cells/experiment, *P<0.05, **P<0.01, and ***P<0.001 by Student’s unpaired t-test or one-way ANOVA with Tukey’s posthoc analysis.

When compared to VEH, basal glucose uptake decreased significantly following the treatment of the myotubes with Lean NEFA or Obese NEFA (F_(3,20)_ = 2.952; *P*<0.05), but there was no difference between VEH and Lean NEFA_(P)_ or between Obese NEFA and Lean NEFA (*P*>0.05; Fig 2A; left). Further analyses revealed significant effect of the type of NEFA treatment on myotybes on GLUT-4 expression (S2A Fig), p-IRS-1 (Tyr895) (S2B Fig) and triacylglycerol accumulation (S2C Fig).

Each NEFA treatment decreased protein synthesis compared to VEH (F_(3,20)_ = 6.994; *P*<0.01), but unlike glucose uptake, the Obese NEFA produced greater suppression of protein synthesis relative to the other NEFA treatments (Fig 2A; right). Treatment with Obese NEFA significantly decreased protein synthesis compared to Lean NEFA or Lean NEFA_(P)_ treatments. A separate group of myotubes were exposed to Obese NEFA as above, but the media was removed following treatment and replaced with normal growth media. After five days of recovery, there was significant increase in glucose uptake (t_(10)_ = 3.759; *P*<0.01) in cells following removal of NEFA (Post) compared with prior to recovery (Pre) (Fig 2B, left). There was no significant difference in protein synthesis following removal of NEFA compared with prior to recovery (*P*>0.05; Fig 2B; right). Gating parameters, representative intensity traces, and scatterplots of each group associated with the above experiments are illustrated in S3 Fig. Scatterplots (S3B Fig) depict the emergence of a distinct population of cells that have approximately the same range of glucose uptake, but shifted towards lower protein synthesis.

### Molecular markers of myogenesis

**Fig 3:**
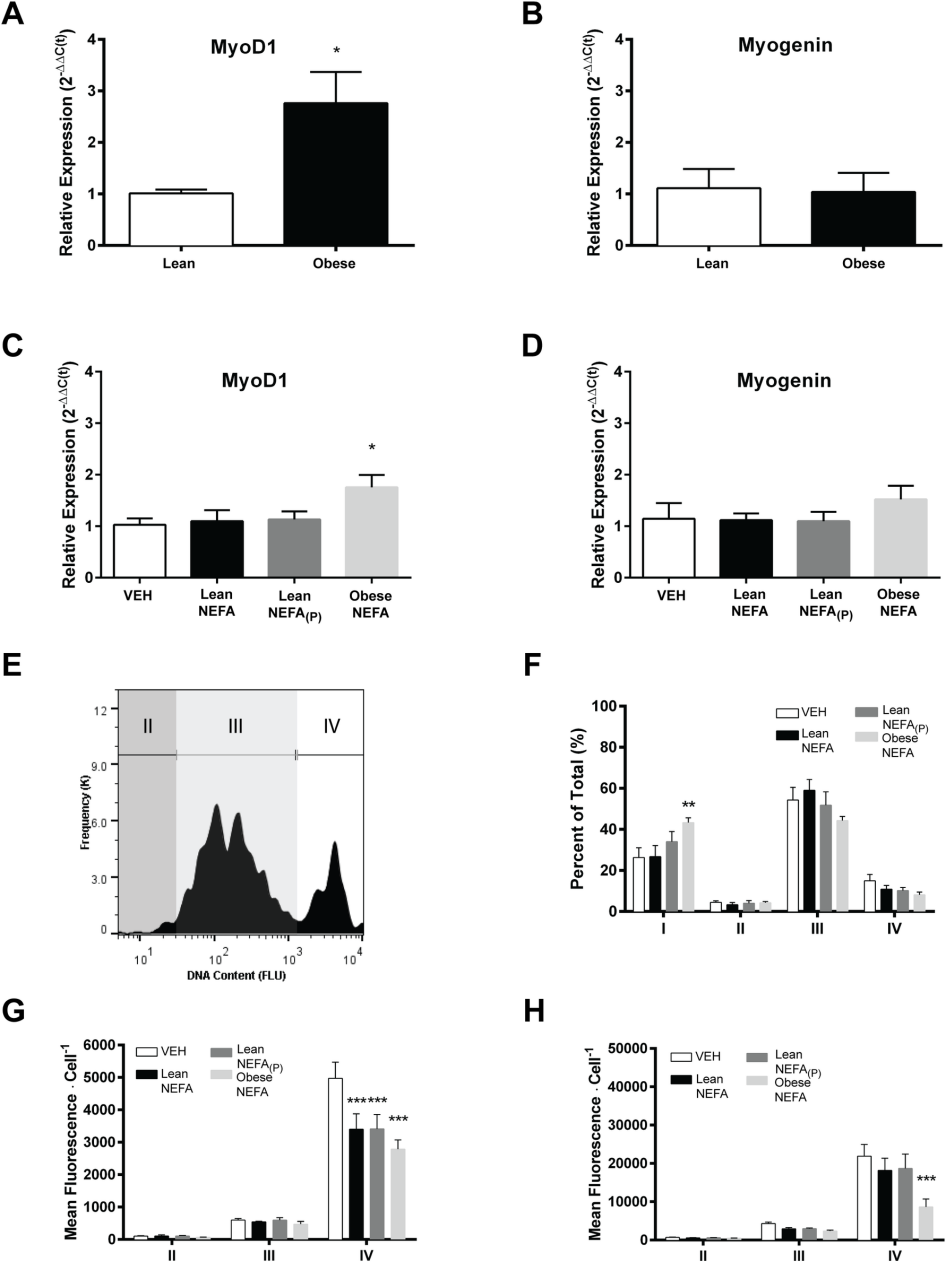
Assessment of growth and differentiation parameters suggest different responses between NEFA exposure groups. (A&C) MyoD1 mRNA was quantified as a marker of myoblast proliferation, and (B&D) myogenin mRNA was used as a marker of myotube differentiation in muscle biopsy or primary culture samples, respectively. (E) A modified fusion index was determined for each cell based on DNA content and categorized based on frequency clusters. (F) Percentages of cells identified in each fusion category were compared. Individual cell populations were analyzed for glucose uptake (G) and nascent protein synthesis (H). Data shown are means ±SE, n = 6 replicates/group, *P<0.05, **P<0.01, and ***P<0.001 by one-way ANOVA with Tukey’s posthoc or two-way ANOVA with Bonferroni’s posthoc analysis.

The molecular markers MyoD1 and myogenin mRNA were used to determine quantitative changes in proliferative myoblasts or differentiating myotubes, respectively. There was a significant 2.763 ± 0.6062 fold increase (t_(11)_ = 2.642; *P* = 0.01) in MyoD1 mRNA expression in skeletal muscle of obese relative to lean subjects (Fig 3A), but no significant difference (t_(11)_ = 0.1344; *P* = 0.4492) in myogenin mRNA expression (Fig 3B). In response to prolonged exposure to serum NEFA, there was significant effect of treatment (F_(3,20)_ = 3.208; *P* = 0.0451) on myoblast proliferation, with Obese NEFA increasing MyoD1 mRNA expression compared to VEH, Lean NEFA and Lean NEFA_(P)_ treatments (Fig 3C). No change was observed with Lean or Lean_(P)_ treatment compared to VEH. There was no significant effect of prolonged exposure to NEFA on myogenin mRNA expression (F_(3,20)_ = 0.7699; *P* = 0.5243; Fig 3D). MyoD1 mRNA is expressed in greater abundance in fast glycolytic muscle compared to fast oxidative or slow oxidative muscle fibers [40,41]. Since obesity is associated with fiber type switching towards fast glycolytic and low oxidative fibers [42], the observed increase in MyoD1 could potentially be due to a fiber type switch, at least *in vivo*. However, MyoD1 also accumulates in non-quiescent satellite cells, such as in regenerating muscle [43], and therefore we further examined the individual cell populations.

### Targeting distinct cell populations

To address possible differences in myogenesis, a fusion index was used to subtype the myotubes based on the degree of differentiation [44], and modified for total DNA content *in lieu* of nuclei quantity. The gating criteria shown in Fig 3E were based on cell frequency clusters at increasing fluorescent (DNA content) intensities, which was consistent across all experiments. Cells that were within the initial size and granulation gate, but were excluded from analysis via the second low glucose/protein synthesis gate, were defined as Type I cells. Population percentages are summarized in Fig 3F. The NEFA treatment only affected Type I cells with Obese NEFA (*P* = 0.0490; F_(9, 60)_ = 2.049) and significantly increased the percentage of Type I cells compared to treatments with VEH and Lean NEFA. Glucose uptake (Fig 3G) and protein synthesis (Fig 3H) were further analyzed per cell population. There was a significant effect of treatment (*P* = 0.0119; F_(3, 20)_ = 4.731), cell type (*P*<0.001; F_(2, 40)_ = 239.1), and interaction (*P*<0.01; F_(6, 40)_ = 4.419). All treatments reduced glucose uptake in Type IV cells when compared to VEH, but there was no significant difference between treatments (*P*>0.05). These results indicate that Type IV cells, with the most nuclear content and metabolically more active, drive the differences in glucose uptake. There was a significant effect of treatment (*P* = 0.0161; F_(3, 20)_ = 4.365), cell type (*P*<0.001; F_(2, 40)_ = 96.80), and interaction (*P* = 0.0146; F_(6, 40)_ = 3.065) on protein synthesis. Specifically, there was lower protein synthesis in Type IV cells treated with Obese NEFA when compared to VEH, Lean NEFA or Lean NEFA_(P)_ treatments. In contrast to glucose uptake, the resolution of treatment group differences in cumulative protein synthesis diminished when separated into individual cell types. Our data suggest that the effects of NEFA treatment on protein synthesis are not specific towards a single population of cells and to the same extent as seen with the glucose uptake.

### β-F1-ATPase protein translation efficiency

**Fig 4:**
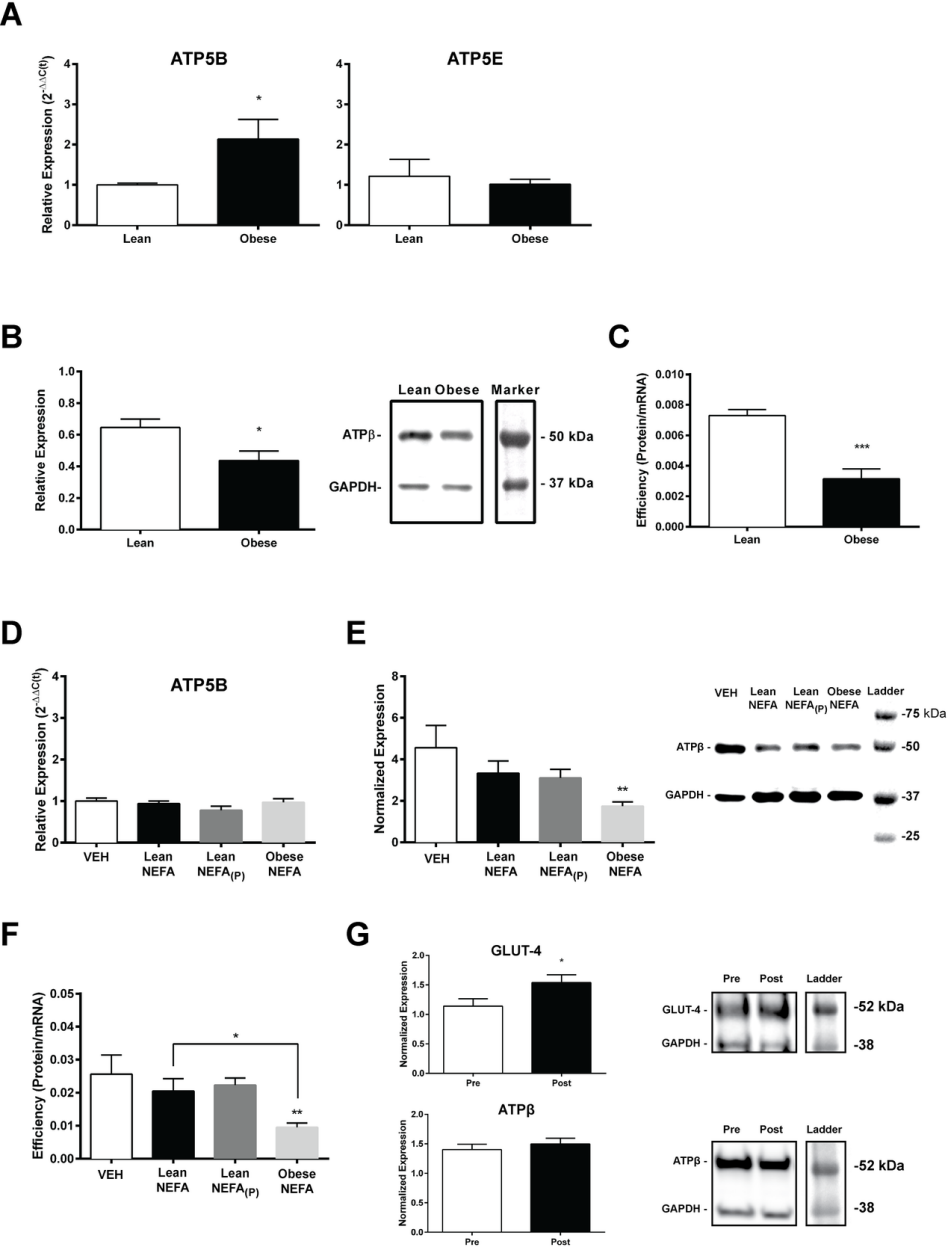
Quantification of *ATP5B* mRNA and β-F1-ATPase protein expression shows decreased protein translation in obesity. (A) The mRNA expression for *ATP5B* and *ATP5E* are shown, respectively. (B) Summary of β-F1-ATPase protein expression and representative blots are depicted. Positive bands for β-F1-ATPase are seen at ~56 kDa and for GAPDH at ~37 kDa. (C) Translation efficiency was compared between lean (n = 6) and obese (n = 7) subjects. Prolonged NEFA exposure on myotube β-F1-ATPase translation was determined in skeletal muscle explants (n = 6/group). (D) *ATP5B* mRNA was quantified for each NEFA-exposed group, (E) differences in β-F1-ATPase protein expression in response to NEFA exposure were observed, and (F) translation efficiency was calculated. (G) An additional myotube group was exposed to Obese NEFA (Pre) and then incubated for an additional five days in normal growth media (Post), and the cells were analyzed for GLUT-4 and β-F1-ATPase protein expressions. Data shown are means ±SE, and significance was *P<0.05, **P<0.01, and ***P<0.001 by Student’s unpaired t-test, or by one-way ANOVA with Tukey’s posthoc analysis.

We focused our attention on β-F1-ATPase to isolate an important protein in energy metabolism that its activity can be influenced by changes in protein abundance. We found a significant (t_(11)_ = 2.121; *P* = 0.0287) 1.83±0.31 fold increase in *ATP5B* mRNA expression in obese individuals compared to lean (Fig 4A; left). In order to determine whether increased mRNA expression is a universal response with respect to mRNAs encoding subunits in the ATP synthase complex in obesity, we evaluated the mRNA expression of *ATP5E*, which encodes the epsilon subunit of the ATP synthase, in a subset of subjects. However, there was no significant difference (t_(11)_ = 0.4496; *P* = 0.3381) in the expression of *ATP5E* mRNA between lean and obese subjects (Fig 4A; right). Calculated Pearson’s product-moment correlation coefficient showed significant correlation between *ATP5B* mRNA and BMI (r = 0.64; *P*<0.01). There was lower β-F1-ATPase protein expression (t_(11)_ = 2.597; *P* = 0.0124) in muscle from obese subjects compared to that in lean controls (Fig 4B). The calculated translation efficiency for β-F1-ATPase was lower in obese subjects compared to the lean subjects (Fig 4C; t_(11)_ = 5.251; *P*<0.001). Long-term repeated exposure of human myotubes to the NEFA treatments, and when compared to VEH, resulted in no difference in *ATP5B* mRNA expression following Lean NEFA, Lean NEFA_(P)_, or Obese NEFA treatments (Fig 4D). The same NEFA treatments, however, resulted in a significant effect on β-F1-ATPase protein expression (F_(3,20)_ = 3.115; *P* = 0.0492; Fig 4E). Posthoc analysis indicated a significant decrease in Obese NEFA-exposed myotubes, but not Lean NEFA or NEFA_(P)_ compared to VEH. There was also a significant difference in β-F1-ATPase protein expression between the two Lean NEFA and Obese NEFA treated groups (*P*<0.05). There was a significant effect of treatment on translation efficiency (F_(3,20)_ = 3.602; *P* = 0.0314; Fig 4F), and posthoc analysis revealed a significant decrease for Obese NEFA-exposed myotubes when compared to VEH, but not for Lean NEFA or Lean NEFA_(P)_. There was a significant difference in translation efficiency between Obese NEFA-exposed myotubes compared to the Lean NEFA treatment (*P* = 0.0473). Removal of the Obese NEFA, although increased GLUT-4 expression (*P* = 0.0259), did not have a significant effect on β-F1-ATPase protein expression (*P* = 0.4910; Fig 4G).

### Translation suppression by miR-127-5p

**Fig 5:**
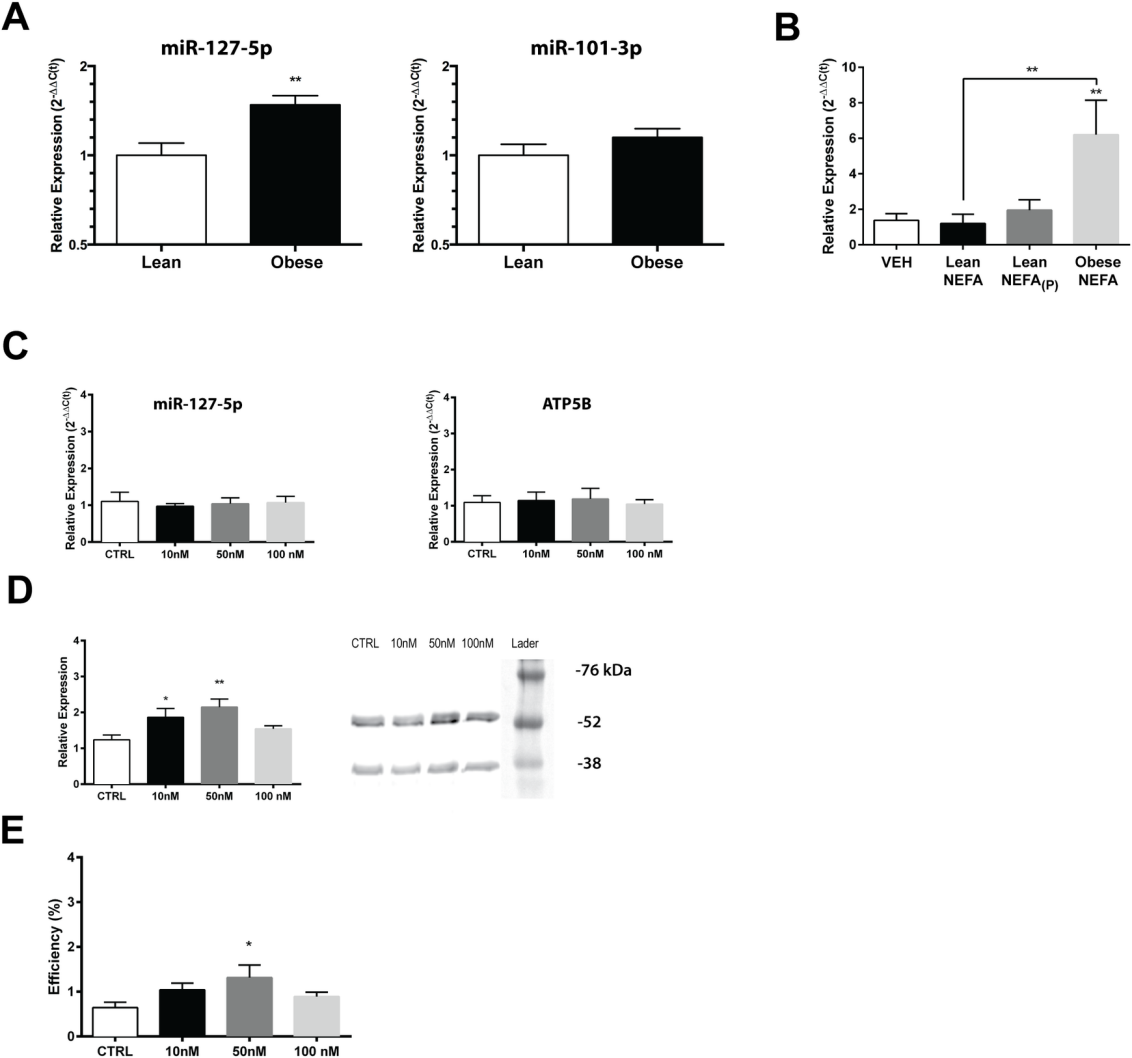
Obesity increases specific skeletal muscle miRNA expression. (A) Expression of miR-127-5p (left) and miR-101-3p (right) were quantified in lean (n = 6) and obese (n = 6) subjects. (B) Changes in myotube miR-127-5p in response to repeated exposure to NEFA extracts were determined (n = 6 replicates/group). Alexa Fluor-488 conjugated locked nucleic acid (LNA) oligos were used to inhibit miR-127-5p in primary myotubes from an obese subject. (C) Response to graded concentrations of inhibitors (10nM-100nM) are shown for miR-127-5p expression and *ATP5B* mRNA expression. (D) Observed responses for β-F1-ATPase protein expression and (E) translation efficiency. Data shown are means ±SE, and significance was *P<0.05 and **P<0.01 by one-way ANOVA with Tukey’s posthoc analysis.

Both miR-101-3p and miR-127-5p have been reported to regulate β-F1-ATPase translation [19,23], and were investigated in the present study as putative intermediates (S4A Fig). There was a significant 1.50±0.01 fold increase in miR-127-5p expression (t_(11)_ = 3.551; *P*<0.01) in skeletal muscle of obese individuals compared to lean controls (Fig 5A), but no difference in miR-101-3p expression (t_(11)_ = 1.273; *P* = 0.1160). The expression of miR-127-5p negatively correlated with β-F1-ATPase protein translation efficiency (r = -0.6744; *P*<0.01). In primary myotubes exposed to NEFA from lean and obese subjects, there was significantly increased expression of miR-127-5p with Obese NEFA treatment when compared to VEH and the two Lean NEFA treatments (F_(3,20)_ = 4.919; *P* = 0.0102), but no difference in Lean NEFA or Lean NEFA_(P)_ treatments when compared to VEH (Fig 5B). To determine the role of miR-127-5p in controlling β-F1-ATPase translation, primary myotubes from an obese individual were exposed to graded concentrations of a miR-127-5p inhibitor. Following transfection, miRNA and mRNA were quantified. Treatment with the inhibitor did not alter either miR-127-5p expression (F_(3,20)_ = 0.09398; *P* = 0.9625) or *ATP5B* mRNA expression (F_(3,20)_ = 0.08332; *P* = 9683) (Fig 5C). There was a significant effect of inhibitor treatment on β-F1-ATPase protein expression (F_(3,20)_ = 4.672; *P* = 0.0125). Exposure to a miR-127-5p inhibitor dose dependently increased β-F1-ATPase protein expression (Fig 5D). There was also a significant effect of miR-127-5p inhibition on β-F1-ATPase translation efficiency (F_(3,20)_ = 4.941; *P* = 0.01; Fig 5E). Compared to control, exposure to 50 nM of inhibitor significantly increased translation. Treatments with 10 nM and 100 nM inhibitor did not reach statistical significance (i.e., *P*<0.05).

### Genomic and epigenomic mechanisms

**Fig 6:**
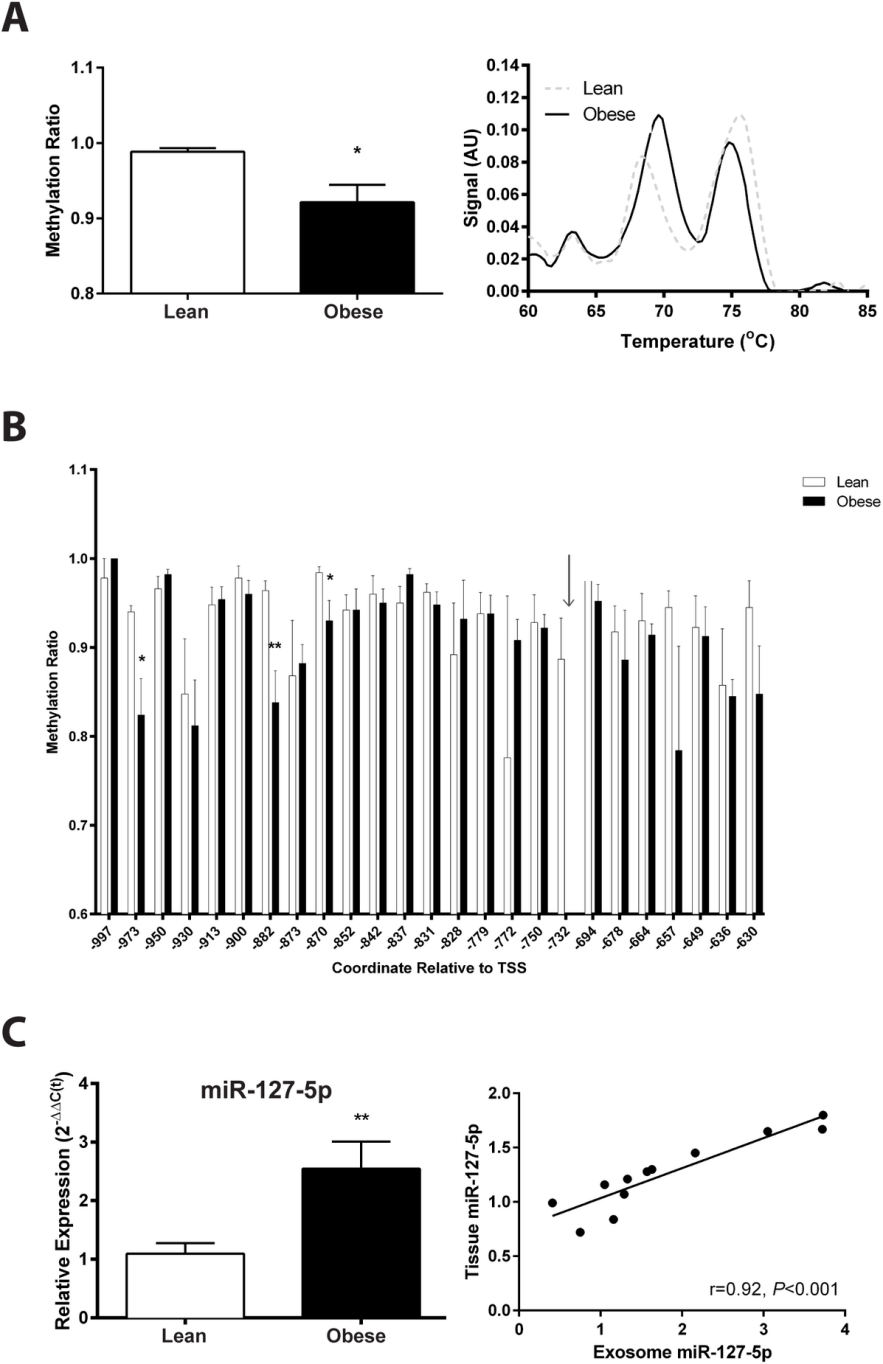
Epigenetic regulation and distribution of miR-127-5p. The 1kB region adjacent to the TSS of miR-127-5p contains four CpG islands, which may be responsible for sustaining expression of the miRNA. (A) High Resolution Melt (HRM) was used to analyze overall DNA methylation status of the 1 kB region upstream of the miR-127-5p TSS in muscle biopsies in the lean (n = 6) and obese (n = 7) subjects. (B) Results from bisulfite sequencing of the first two CpG islands of the 1 kB region identified 26 methylation sites in lean (n = 5) and obese (n = 5) subjects. No results could be obtained for obese subjects at the site indicated by the arrow. (C) Total RNA was extracted from serum exosomes of lean (n = 6) and obese (n = 6) subjects, analyzed for miR-127-5p expression (left), and correlation with skeletal muscle expression was determined (right). Data shown are means ±SE, *P<0.05 and **P<0.01 by Student’s unpaired t-test.

The 1 kB region adjacent to the TSS of miRNAs is known to be important for transcription initiation and is CpG-rich. The methylation status of the 1 kb region was analyzed via high-resolution melt (Fig 6A), and revealed a significant decrease (t_(11)_ = 2.632; *P* = 0.0117) in the proportion of methylated CpGs in DNA extracted from the skeletal muscle of obese subjects compared to those of lean controls. We further analyzed the DNA methylation profile of the first two CpG islands via bisulfite sequencing (S5 Fig). Overall, there was a significant effect of obesity (F_(1, 186)_ = 19.05; *P*<0.001), CpG site (F_(24, 186)_ = 9.698; *P*<0.001), and interaction (F_(24, 186)_ = 7.240; *P*<0.001). Posthoc analysis identified three of the methylation sites that were significantly decreased in the skeletal muscle of obese subjects compared to lean: TSS -973 (11.60±6.371%), -882 (12.60±6.007%), -870 (5.400±6.007%) (Fig 6B). The methylation analysis failed for all obese subjects at site TSS -732, and further review of individual chromatograms identified a C→G single nucleotide polymorphism (SNP; rs11623267; S6A Fig), which eliminates cysteine methylation at that site. There is a paucity of information regarding the precise effect of hypomethylation on miRNA expression, since miRNA ‘promoters’ are generally indistinguishable from coding gene promoters [45]. However, the miRNA control region can be characterized by H3K4me3 occupation of a methylated CpG island within 1 kb of the TSS [46]. Histone binding of this region, in addition to methylation of the DNA, suppresses transcription and expression of the miRNAs. Moreover, transcription factor consensus sequence analysis identified binding sites for the histone-modifying complex CBP/p300 at all significantly altered methylation sites and the SNP site (S6B Fig).

To expand upon potential clinical applicability of these findings, exosomes extracted from patient sera collected at the time of the muscle biopsies were analyzed to determine whether miR-127-5p is present in exosomes and the degree of correlation with skeletal muscle miR-127-5p expression. Analysis of the small RNAs present in the exosomes revealed the presence of miR-127-5p (S7C Fig). To determine if the exosomes originated at least partially from muscle tissue, the extracts were probed for Desmin and GAPDH and resulted in positive signals (S7D Fig). Furthermore, we found that exosomes extracted from the media of the primary myotube cultures also expressed miR-127-5p (S7E Fig), further supporting a link between exosomal miR-127-5p and muscle miRNA. Upon further analysis of miR-127-5p expression in the exosomes, we found a significant 2.547±0.4609 fold increase (t_(11)_ = 2.934; *P*<0.01) in obese subjects compared to lean controls (Fig 6C). The expression of miR-127-5p in exosomes significantly correlated with skeletal muscle miR-127-5p content (r = 0.9184; *P*<0.001).

## Discussion

We provide in-depth mechanistic insights into how obesity perturbates skeletal muscle protein metabolism as it relates to the β-F1-ATPase, and advancing previous findings on mitochondrial protein metabolism in obesity [3]. Our data suggest that this response in the muscle of obese individuals is linked specifically to the plasma NEFA profile associated with obese phenotype. Furthermore, our findings implicate certain epigenetic mechanisms involving miRNA regulation in sustaining the impaired translation of muscle β-F1-ATPase involved in energy metabolism. These innovative findings establish direct effects of plasma NEFA on impairing the metabolism of β-F1-ATPase, open new avenues of research, and have clinical implications for understanding mechanisms underlying metabolic dysfunction in muscle (i.e., impaired muscle ATP production [47]).

There is large variability in protein expression in muscle of obese compared to lean individuals at the single-protein level in general, and mitochondrial level in particular [9]. By focusing on a single mitochondrial protein and using a series of experiments, we were able to unravel mechanisms by which circulating NEFA associated with obese phenotype suppress the metabolism of β-F1-ATPase (summarized in S8 Fig). Our human data, obtained in a limited number of subjects, may not be representative of muscle protein metabolism across lean and obese subjects. However, lower total muscle protein synthesis in obese when compared to lean controls in our study is in line with previous findings in humans [3]. Also, and comparable to earlier findings in humans [12,48], β-F1-ATPase was lower in muscle from the obese subjects. When considering the overall lower rate of synthesis of mitochondrial proteins in obesity [3], together with the reduced translation efficiency of β-F1-ATPase in the obese subjects in the present studies, it is reasonable to conclude that the rate of synthesis of muscle β-F1-ATPase is impaired in obesity.

Our findings suggest that a key element contributing to suppressed β-F1-ATPase metabolism in muscle is chronic exposure to lipids associated with the obese phenotype. In the *in vivo* circumstance, these lipids may include plasma NEFA, fatty acids liberated by the action of lipoprotein lipase in circulating triacylglycerols, or fatty acids released within tissue after stored as triacylglycerols in skeletal muscle. Regardless of the source of fatty acids, changes in skeletal muscle protein metabolism may be secondary to initial changes in the plasma lipid milieu. Undoubtedly, much evidence implicates excessive circulating NEFA in skeletal muscle ectopic lipid accumulation and lipotoxicity that in turn results in insulin resistance [49,50], and impaired muscle protein metabolism [32]. However, it is important to note that elevated plasma NEFA levels alone may not always impair muscle protein synthesis [6], and that the composition of fatty acids, as documented in the present studies, may be of greater importance in disrupting muscle protein metabolism. Importantly, circulating lipids associated specifically with obesity induce aberrant protein synthesis and glucose metabolism in muscle via different mechanisms. We observed a different pattern in glucose uptake versus protein synthesis following NEFA treatment, and found that removal of NEFA did not recover total protein synthesis or β-F1-ATPase protein abundance in myotubes despite restoring muscle glucose uptake. Thus, the effects of NEFA exposure on muscle protein metabolism are more persistent than those on glucose metabolism. Our findings offer a potential explanation as to why, unlike improvements in plasma glucose homeostasis, β-F1-ATPase protein abundance was not restored by exercise intervention [48].

Unlike the epsilon subunit of the ATP synthase complex, the beta subunit of the ATP synthase was differentially regulated between lean and obese subjects at the mRNA expression level. Our results implicate NEFA-induced miR-127-5p expression in suppressing muscle β-F1-ATPase translation in obesity, despite an increase in β-F1-ATPase mRNA expression in the obese subjects. As an extension of the present study, the quantification of miR-127-5p in plasma exosomes provides a viable marker of β-F1-ATPase metabolism. Hypomethylation of the miR-127-5p control region emerged as an epigenetic mechanism contributing to the chronicity of reduced muscle β-F1-ATPase translation in obesity. We identified decreased site-specific methylation in consensus sequences associated with histone-modifying elements, linking our data to an epigenetic mechanism, which is, however, beyond the scope of this study. Within the same region, our analysis also revealed a SNP in all obese subjects, removing a cytosine methylation site completely. These findings illustrate a gene-environment interaction that may be a key mechanism sustaining the increased miR-127-5p expression and subsequently decreased β-F1-ATPase translation in obesity.

A key strength of our *in vitro* experiments was the approach of exposing primary myotubes to the precise distribution of plasma NEFA characteristic of obese individuals. Additionally, by using NEFA concentrations comparable to those observed *in vivo* for relatively longer period of time, our treatment more precisely modeled the obese condition. Therefore, our series of experiments can link directly *ex vivo* observations to the *in vivo* regulation of muscle protein metabolism in obesity. In concurrence with a recent epidemiological study [51], our results implicate the distribution of PUFA versus SFA (i.e., PUFA/SFA ratio) rather than total lipid concentration in the regulation of muscle protein metabolism.

A recent study reported that combined increase in plasma insulin and amino acid concentrations sufficiently stimulates protein synthesis of myofibrillar and sarcoplasmic proteins, representing the bulk of proteins in muscle, in obese individuals when compared to lean controls [52]. However, hyperinsulinemia/hyperaminoacidemia did not increase the synthesis of muscle mitochondrial proteins in obesity [3]. Indeed, as detailed in our experiments, increasing the abundance of mitochondrial proteins, such as β-F1-ATPase, may be resistant to traditional interventions due to epigenetic mechanisms activated in obesity via plasma NEFA. Further investigation into these mechanisms becomes critical in devising scientifically sound interventions that reverse the persistent effects of obesity on muscle protein metabolism.

We provide in-depth, detailed mechanistic evidence linking plasma NEFA associated with obese phenotype to suppressed muscle β-F1-ATPase metabolism. These findings extrapolated from the cell culture model show that the plasma NEFA may play a role in impairing the abundance of muscle β-F1-ATPase in obesity. These effects appear to be independent of the role of NEFA in impairing muscle glucose metabolism.

## Supporting Information

S1 Appendix Experimental Procedures. (DOCX)

S1 Dataset (XLSX)

S1 Fig No qualitative changes in growth and differentiation of skeletal muscle explants in response to NEFA exposure. (A) Myoblast cultures were propagated from skeletal muscle biopsies, and differentiation of myoblasts to nascent myotubes and mature myotubes was induced. Myotube differentiation was initiated after six days and continued until NEFA exposure initiation on day 16. NEFA were supplemented every other day for one week. (B) Representative photomicrographs are shown for each group at the end of the NEFA exposure period. Scale bar represents 500 μm.
(TIFF)

S2 Fig NEFA exposure alters markers of insulin resistance in culture myotubes. (A) GLUT-4 protein expression. (B) Phosphorylated IRS-1 (Tyr895) expression. (C) Intracellular triacylglycerol concentration. (D) Free fatty acid content of the culture media at the end of experimentation. Data shown are means ±SE, n = 6 replicates/group, and significance was *P<0.05, **P<0.01, and ***P<0.001 by one-way ANOVA with Tukey’s posthoc analysis.
(TIFF)

S3 Fig Concurrent assessment of glucose uptake and nascent protein synthesis in primary human myotubes. Fluorescent glucose (2-NBDG) and O-propargyl-puromycin (OPP) incorporation in primary human myotubes was quantified by flow cytometry. (A) Gating parameters and representative intensity traces. (B) A scatterplot of all cells (black), VEH (red), Lean NEFA (orange), Lean NEFA_(P)_ (blue), Obese NEFA (green), and recovery group (grey).
(TIF)

S4 Fig Obesity and skeletal muscle miRNA expression implicated in regulation of β-F1-ATPase protein. (A) Both miR-101-3p (left) and miR-127-5p (right) can regulate β-F1-ATPase protein translation (microrna.org). (B) Brightfield photomicrographs and fluorescent photomicrographs are shown for the treatment concentrations associated with the data presented in Fig 5.
(TIF)

S5 Fig miR-127-5p. A schematic diagram of the CpG islands of the 1 kb region for miR-127-5p. The 1kB region adjacent to the TSS of miR-127-5p contains four CpG islands, which may be responsible for sustaining expression of the miRNA.
(TIF)

S6 Fig Analysis of the miR-127-5p promoter region. Transcription factor binding analysis revealed consensus sequences for the C/EBP (bold) and p300 (underlined) complex at all significantly decreased methylation sites indicated with an asterisk (*). The representative sequencing chromatogram depicts the cause for the lack of methylation data from obese subjects at the -732 site was due to a SNP (rs11623267).
(TIFF)

S7 Fig Control experiments. (A) LC-MS/MS verification of β-F1-ATPase protein in the ~50 kDa band precipitated by the β-F1-ATPase antibody. (B) Bioanalyzer results of RNA ladder (left) and total RNA extracted from exosomes (right) showing presence of small RNAs. (C) Amplification curve, melting curve, and DNA agarose gel for qRT-PCR analysis of hsa-miR-127-5p expression in serum exosomes. (D) Total proteins extracted and Western blot analysis of desmin (~50 kDa), GAPDH (~37 kDa), and CD-9 (~27 kDa) from serum exosomes. (E) DNA agarose gel, amplification curve, and melting curve for qRT-PCR analysis of hsa-miR-127-5p expression in exosomes extracted from myotube culture media.
(TIFF)

S8 Fig Model of β-F1-ATPase Translation Regulation in Obesity. Genetic and epigenetic factors unmask transcription of miR-127-5p, which can then bind to the 3’ UTR of the *ATP5B* mRNA and block ribosomal translation machinery from accessing the transcript.
(TIF)

S9 Fig Western blots. Original, uncropped, and unadjusted western blots for the data presented in the Figures in the Results Section.
(TIF)
