# Supplementary material for: Prolonged Exposure of Primary Human Muscle Cells to Plasma Fatty Acids Associated with Obese Phenotype Induces Persistent Suppression of Muscle Mitochondrial ATP Synthase β Subunit
Source: PLoS One. 2016 Aug 17;11(8):e0160057. doi: 10.1371/journal.pone.0160057 (PMC4988792; doi:10.1371/journal.pone.0160057)
Supplement: S1 Appendix — (DOCX) [file pone.0160057.s001.docx]

**Supporting Material - Experimental Procedures**

*Primary Tissue Culture*

Skeletal muscle tissue was collected in low-glucose (1 g/L) DMEM with sodium pyruvate (1 mM), Penicillin-Streptomycin (25,000 U/mL) and supplemented with 16% heat-inactivated Cosmic Calf Serum (GE Healthcare Life Sciences, Pittsburg, PA). The tissue was dissociated with 0.25% Trypsin-EDTA/0.068% Collagenase IV and initially plated on non-coated flasks to remove fibroblasts, and subsequently on collagen-coated flasks (Corning Inc. Life Sciences, Tewksbury, MA). The growth media was supplemented with dexamethasone (1 nM; MP Biomedicals LLC, Santa Ana, CA), insulin (1 μM; Life Technologies, Carlsbad, CA), and hEGF (1 ng/μL; Life Technologies, Carlsbad, CA). Differentiation of myoblasts to myotubes was induced with high glucose (4.5 g/L) DMEM supplemented with GlutaMAX, growth factors, and 10% Cosmic Calf Serum. Following differentiation, myotubes were left undisturbed for an additional 48 hrs before experimentation. Tissue culture photomicrographs were contrast inverted, and a PFX 15x filter was applied using Picfx software (ver. 6.0.6, Active Development Ltd., London, UK).

*Non-Esterified Fatty Acid Extraction*

Total non-esterified fatty acids (NEFA) were derived from select subject sera for myotube treatments. NEFA were extracted using LC-NH_2_ Columns (Sigma-Aldrich, Hershey, MA). Lipids were separated with chloroform and bound to the hexane-conditioned LC-NH_2_ columns. The columns were washed with chloroform and cleared of neutral lipids with chloroform/isopropanol (2:1). The NEFA were eluted with 2% acetic acid/diethylether, and the solvents were evaporated with a continuous stream of nitrogen gas. The NEFA were then reconstituted in Cosmic Calf Serum with BSA (20 mg/mL) as a carrier. Cosmic Calf Serum + NEFA were diluted to reflect extracellular skeletal muscle conditions in the primary tissue culture experiments. Final NEFA concentrations were determined using an enzymatic, calorimetric quantification method (Wako Chemicals, Richmond, VA), and final lipid panel after dilution in media was confirmed via LC-MS/MS [[1](#_ENREF_1)].

*Quantification of mRNA/miRNA*

Total RNA was extracted from approximately 25 mg of muscle using QIAzol lysis reagent and column purified (Qiagen Inc., Valencia, CA). The QuantiTect system was used for first strand synthesis and qRT-PCR with predesigned QuantiTect Primer assays for *ATP5B*, *ATP5E*, MYOD1, MYOG, GAPDH, and ACTB. Expression of ACTB was determined to be inconsistent and varied with age, similar to our previous report [[2](#_ENREF_2)]. Thus, mRNA levels in the sample were normalized to GAPDH using the comparative (2^–ΔΔCT^) method. Selected miRNAs were quantified according to Tran and Greenwood-Van Meerveld [[2](#_ENREF_2)]. Briefly, cDNA was generated for mature miRNAs using the miScript II RT kit. The QuantiTect system was used for qRT-PCR as described above with predesigned QuantiTect primers for hsa-miR-101-3p and hsa-miR-127-5p. Expression of miRNAs was normalized to RNU-6B.

*Protein Quantification*

Total DNA was extracted from the organic/interphase with EtOH, and proteins were precipitated from the remaining phenol solution with isopropanol. The proteins were pelleted and washed with 0.3M guanidine-HCl/95% EtOH. The pellet was resuspended with equal volumes of 1% SDS-Tris HCl/ 8 M Urea and briefly sonicated to dissolve. Insoluble material was pelleted and the protein-containing supernatant was diluted with RIPA buffer. Detection of proteins of interest was performed by Western blot according to standard protocols [[2-4](#_ENREF_2)]. Approximately 30 μg of protein were resolved on an SDS-PAGE 4-30% linear gradient gel, transferred to a nitrocellulose membrane, blocked, and probed with respective antibodies. Positive bands were visualized using Clarity^TM^ Western ECL kit (Bio-Rad, Hershey, PA) and normalized to GAPDH. Antibodies used in this study included anti-β-F1-ATPase (Abcam, Cambridge, MA), anti-GAPDH (Rockland, Inc., Gilbertsville, PA), anti-GLUT4 (Millipore, Billerica, MA), anti-phospho-IRS-1 (Tyr895) (Life Technologies, Carlsbad, CA), anti-Desmin (Santa Cruz Biotechnology, Inc.), anti-CD9 (SBI Systems Biosciences). Secondary anti-rabbit and anti-mouse were purchased from Santa Cruz Biotechnologies (Santa Cruz, CA). Positive identification of β-F1-ATPase was confirmed by extracting the corresponding band following SDS-PAGE and analyzing by LC-MS/MS (Figure S7A). Efficiency was calculated following the method of Stevens and Brown [[5](#_ENREF_5)], where Efficiency = Protein x (1-e^-kdeg^)/RNA level. The kdeg for β-F1-ATPase was reported as 0.009662711.

*Determination of Glucose Uptake and Protein Synthesis in Cell Culture*

The culture media was replaced with low glucose (1 g/L) DMEM supplemented with 10% dialyzed fetal bovine serum. After one hour, the media was exchanged with differentiation media containing the fluorescent glucose analogue 2-deoxy-2-[(7-nitro-2,1,3-benzoxadiazol-4-yl) amino]-D-glucose) (2-NBDG; Sigma-Aldrich, St. Louis, MO), the cell-permeable puromycin analogue O-propargyl-puromycin (OPP; Life Technologies, Carlsbad, CA). The OPP is incorporated into newly synthesized polypeptides and can be used to detect nascent protein synthesis. Following a 1-hr incubation, the cells were processed with the Click-iT Plus OPP Alexa Fluor 594 Protein Synthesis Assay kit, and the HCS NuclearMask Blue stain (Life Technologies, Carlsbad, CA) was used to quantify DNA content. The cells were analyzed using a CyAn ADP Analyzer (Beckman Coulter, Inc., Brea, CA) with set filters 488, 594, and 405 to detect 2-NBDG, OPP, and HCS NuclearMask Blue stain respectively. The data was analyzed with FlowJo software (FlowJo, LLC, Ashland, OR) using gating parameters described by Zheng et al. [[6](#_ENREF_6)]. A second gate was applied to exclude cells with low glucose uptake and low protein synthesis.

*Inhibition of miR-127-5p in Cell Culture*

Locked nucleic acid (LNA) inhibitors (Exiqon, Woburn, MA) were synthesized to target miR-127-5p. The LNA inhibitors were conjugated to Alexa Flour 488 for detection of transfection efficiency, and transfection was carried out using Lipofectamine® RNAiMAX (Life Technologies Carlsbad, CA). Media was removed and replaced with Opti-MEM (Life Technologies, Carlsbad, CA). Inhibitors and transfection reagent were combined according to the manufacturer’s instructions at concentrations of 10, 50, and 100 nM of inhibitor. A random oligonucleotide sequence was used as a negative control. Approximately 24 hrs following transfection, the cells were harvested with QIAzol lysis reagent for subsequent RNA and protein detection as described above.

*Exosome Isolation from Serum*

Serum samples were collected at the time of the muscle biopsies, and exosomes were subsequently precipitated with ExoQuick reagent (Systems Biosciences, Inc., Mountain View, CA) following the manufacturer’s protocol. The exosome pellet was washed, resuspended in QIAzol lysis reagent, and miRNAs were extracted using the miRNeasy kit. The RNA quality was verified on the Agilent Bioanalyzer (Figure S7B). Detection of miR-127-5p proceeded as described above. Amplification curves, melting curves, and DNA gels for the PCR products were performed as controls (Figure S7C). Alternatively, the exosome pellet was resuspended in RIPA buffer, diluted with Lammeli Sample Buffer and analyzed via Western blot.

*DNA Methylation*

DNA methylation was assessed using the sodium bisulfite sequencing method previously described [[7](#_ENREF_7)]. Total DNA collected from the phenol/chloroform extraction was purified and treated with sodium bisulfite using the EpiTect Bisulfite kit (Qiagen Inc., Valencia, CA). The 1 kb region proximal to the transcription start site for miR-127-5p was amplified by nested PCR using the following outer primers: fwd 5’-TTT TTT TTA TYG GAG GTT TTT TAT AGG TGG TAG-3’, rev 5’-TTT ACR CTC TCC CCA AAC CCT ATC ATA CCT C-3’ (903 bp), and inner primers: fwd 5’- TTT TTG TGT TGA GAA GGA TTA TGA T -3’, rev 5’- ACC CCT AAC TCC AAA CTA TCT CTA C -3’ (454 bp). The cycling conditions were 94^o^C for 10 min followed by 40 cycles of 94^o^C for 15s, 55^o^C for 45s, and 72^o^C for 45s, and touchdown annealing temperatures for the first 15 cycles. The products were then purified and sequenced with the inner fwd primer. Proportion of methylation on each CpG site was detected using the Epigenetic Sequencing Methylation analysis software (ESME). The DNA sample from one obese subject failed to sequence.

*Fractional Synthesis Rates (FSR) Calculation in Human Experiments*

The fractional synthesis rate (FSR; %∙hour^-1^) of total muscle protein was calculated as follows:

$$\frac{\Delta E_{m}}{E_{b}\cdot T} \cdot60 \cdot100\%$$

where ΔE_m_ represents the increment in the muscle protein-bound [^13^C_6_]phe enrichment between the muscle biopsies, E_b_ is the average blood [^13^C_6_]phe enrichment in the collective time points between the muscle biopsies, T is the time interval (min) between the biopsies, and the factors 60 and 100 are used to express the FSR values in %∙hour^-1^.

**REFERENCES**

**1.** Persson XM, Blachnio-Zabielska AU, Jensen MD. Rapid measurement of plasma free fatty acid concentration and isotopic enrichment using LC/MS. J Lipid Res. 2010;51: 2761-2765.

2. Tran L, Greenwood-Van Meerveld B. Age-associated remodeling of the intestinal epithelial barrier. J Gerontol A Biol Sci Med Sci. 2013;68: 1045-1056.

3. Tran L, Schulkin J, Greenwood-Van Meerveld B. Importance of CRF receptor-mediated mechanisms of the bed nucleus of the stria terminalis in the processing of anxiety and pain. Neuropsychopharmacology. 2014;39: 2633-2645.

4. Tran L, Schulkin J, Ligon CO, Greenwood-Van Meerveld B. Epigenetic modulation of chronic anxiety and pain by histone deacetylation. Mol Psychiatry. 2015;20: 1219-1231.

5. Stevens SG, Brown CM. In silico estimation of translation efficiency in human cell lines: potential evidence for widespread translational control. PLoS One. 2013;8: e57625.

6. Zheng B, Cao B, Crisan M, Sun B, Li G, Logar A, et al. Prospective identification of myogenic endothelial cells in human skeletal muscle. Nat Biotechnol. 2007;25: 1025-1034.

7. Tran L, Chaloner A, Sawalha AH, Greenwood Van-Meerveld B. Importance of epigenetic mechanisms in visceral pain induced by chronic water avoidance stress. Psychoneuroendocrinology. 2013;38: 898-906.
