# Supplementary figures and images for: Prolonged Exposure of Primary Human Muscle Cells to Plasma Fatty Acids Associated with Obese Phenotype Induces Persistent Suppression of Muscle Mitochondrial ATP Synthase β Subunit
Source: PLoS One. 2016 Aug 17;11(8):e0160057. doi: 10.1371/journal.pone.0160057 (PMC4988792; doi:10.1371/journal.pone.0160057)

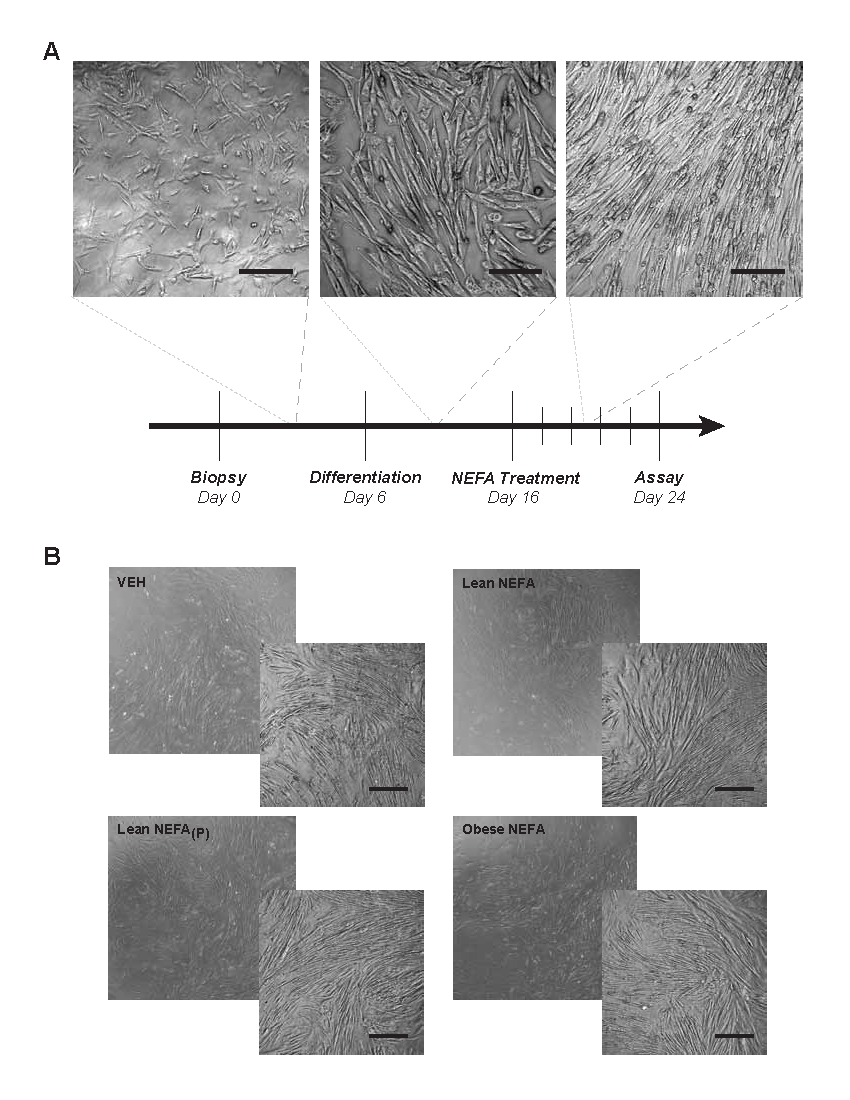

Supplement: S1 Fig — (A) Myoblast cultures were propagated from skeletal muscle biopsies, and differentiation of myoblasts to nascent myotubes and mature myotubes was induced. Myotube differentiation was initiated after six days and continued until NEFA exposure initiation on day 16. NEFA were supplemented every other day for one week. (B) Representative photomicrographs are shown for each group at the end of the NEFA exposure period. Scale bar represents 500 μm. (TIFF) [file pone.0160057.s003.tiff]

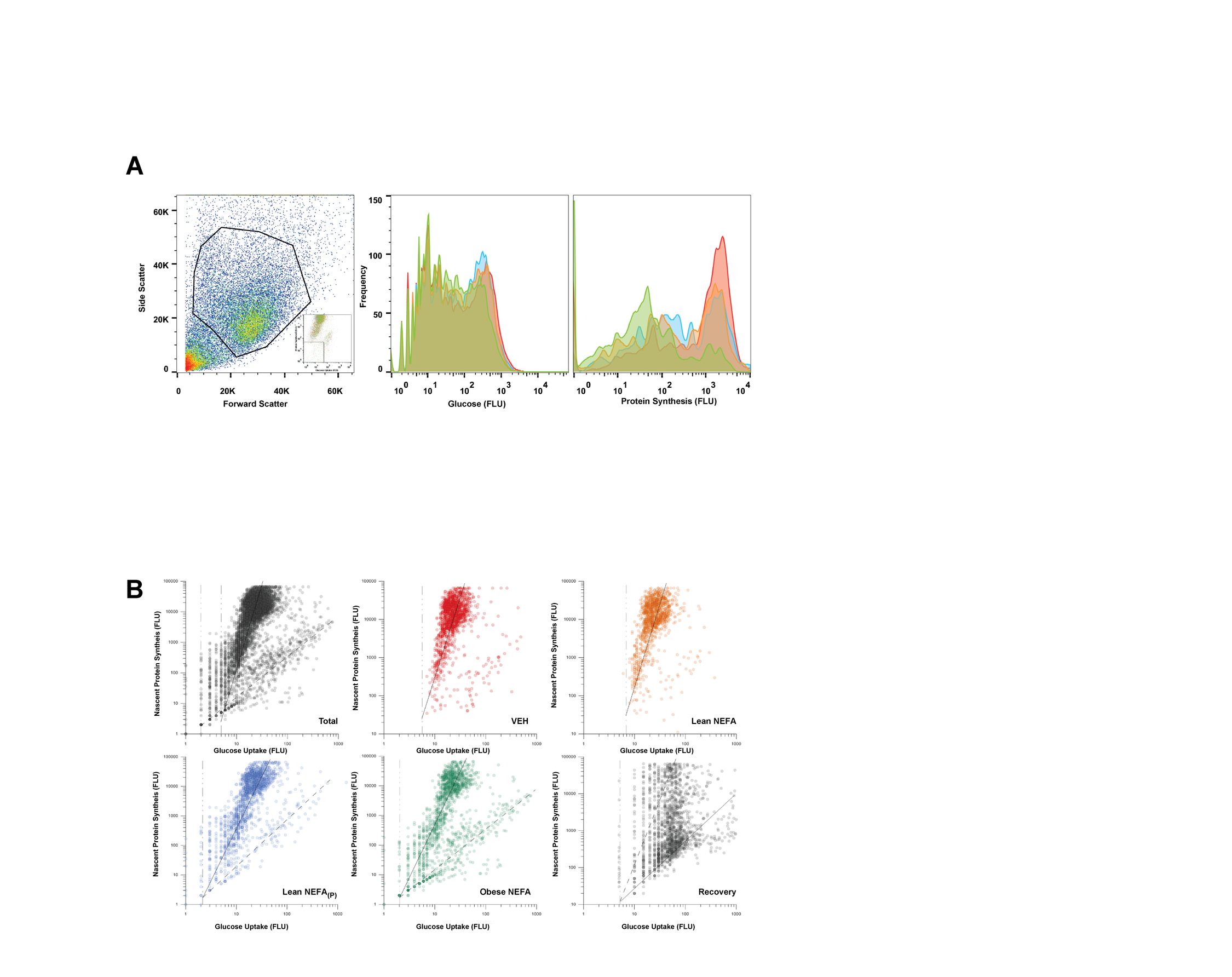

Supplement: S3 Fig — Fluorescent glucose (2-NBDG) and O-propargyl-puromycin (OPP) incorporation in primary human myotubes was quantified by flow cytometry. (A) Gating parameters and representative intensity traces. (B) A scatterplot of all cells (black), VEH (red), Lean NEFA (orange), Lean NEFA(P) (blue), Obese NEFA (green), and recovery group (grey). (TIF) [file pone.0160057.s005.tif]

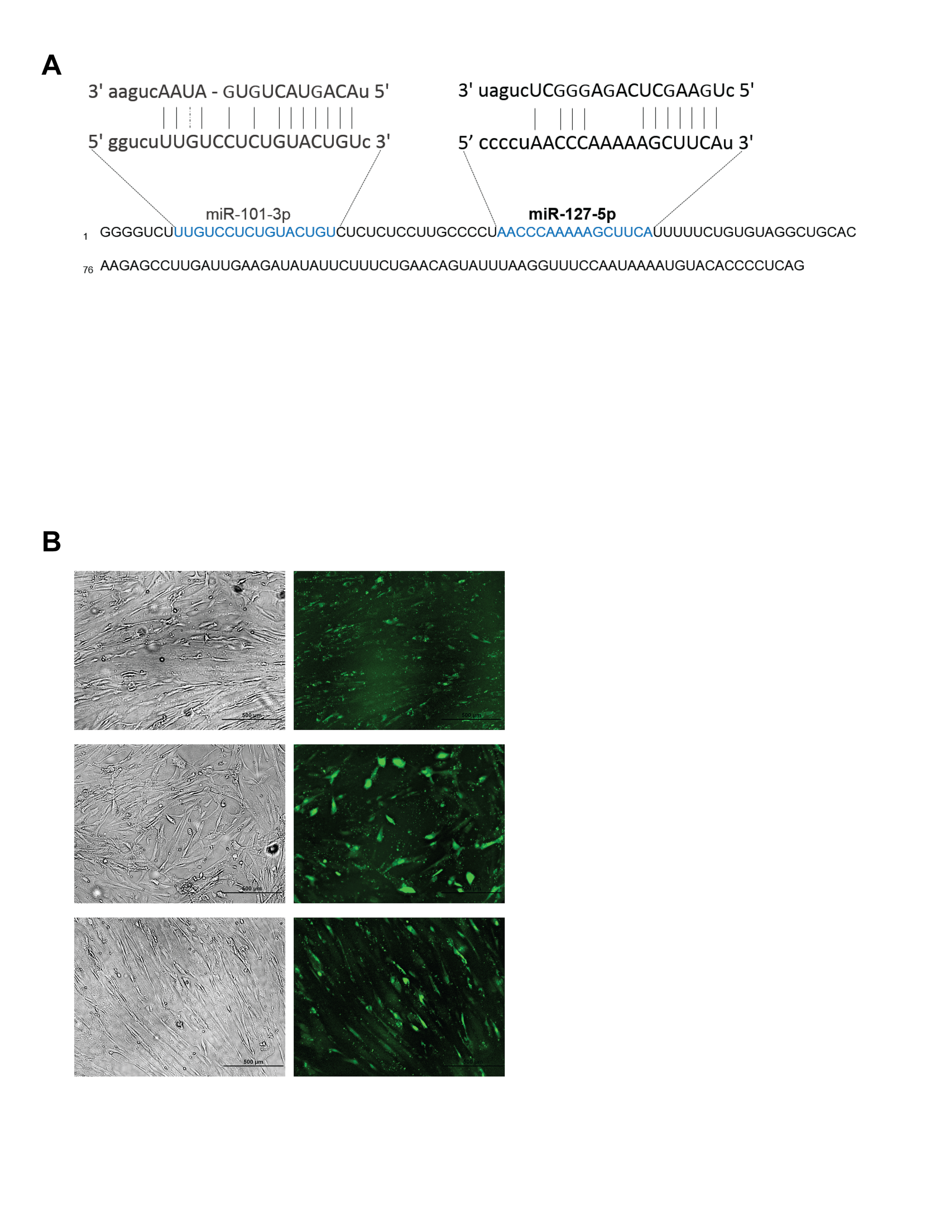

Supplement: S4 Fig — (A) Both miR-101-3p (left) and miR-127-5p (right) can regulate β-F1-ATPase protein translation (microrna.org). (B) Brightfield photomicrographs and fluorescent photomicrographs are shown for the treatment concentrations associated with the data presented in Fig 5. (TIF) [file pone.0160057.s006.tif]

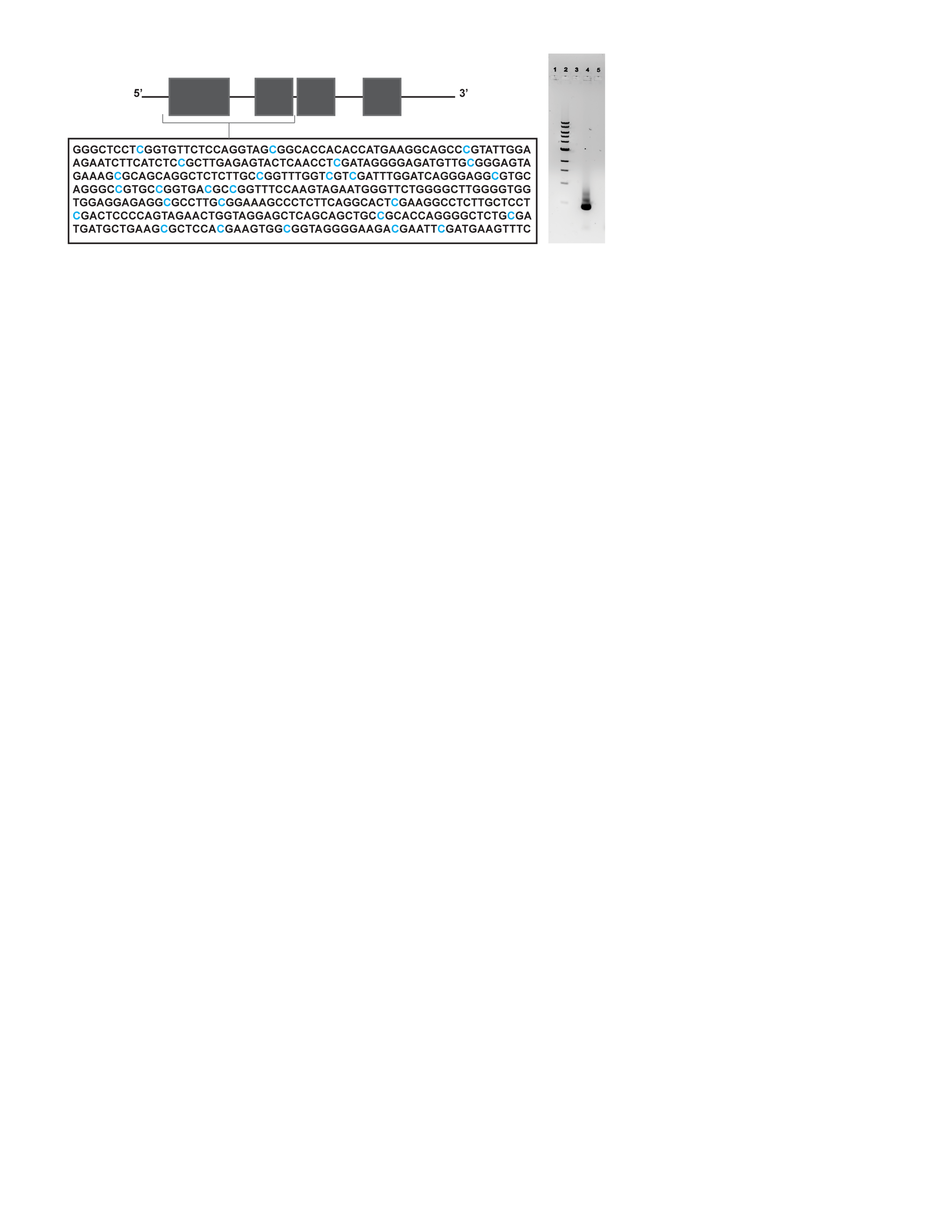

Supplement: S5 Fig — A schematic diagram of the CpG islands of the 1 kb region for miR-127-5p. The 1kB region adjacent to the TSS of miR-127-5p contains four CpG islands, which may be responsible for sustaining expression of the miRNA. (TIF) [file pone.0160057.s007.tif]

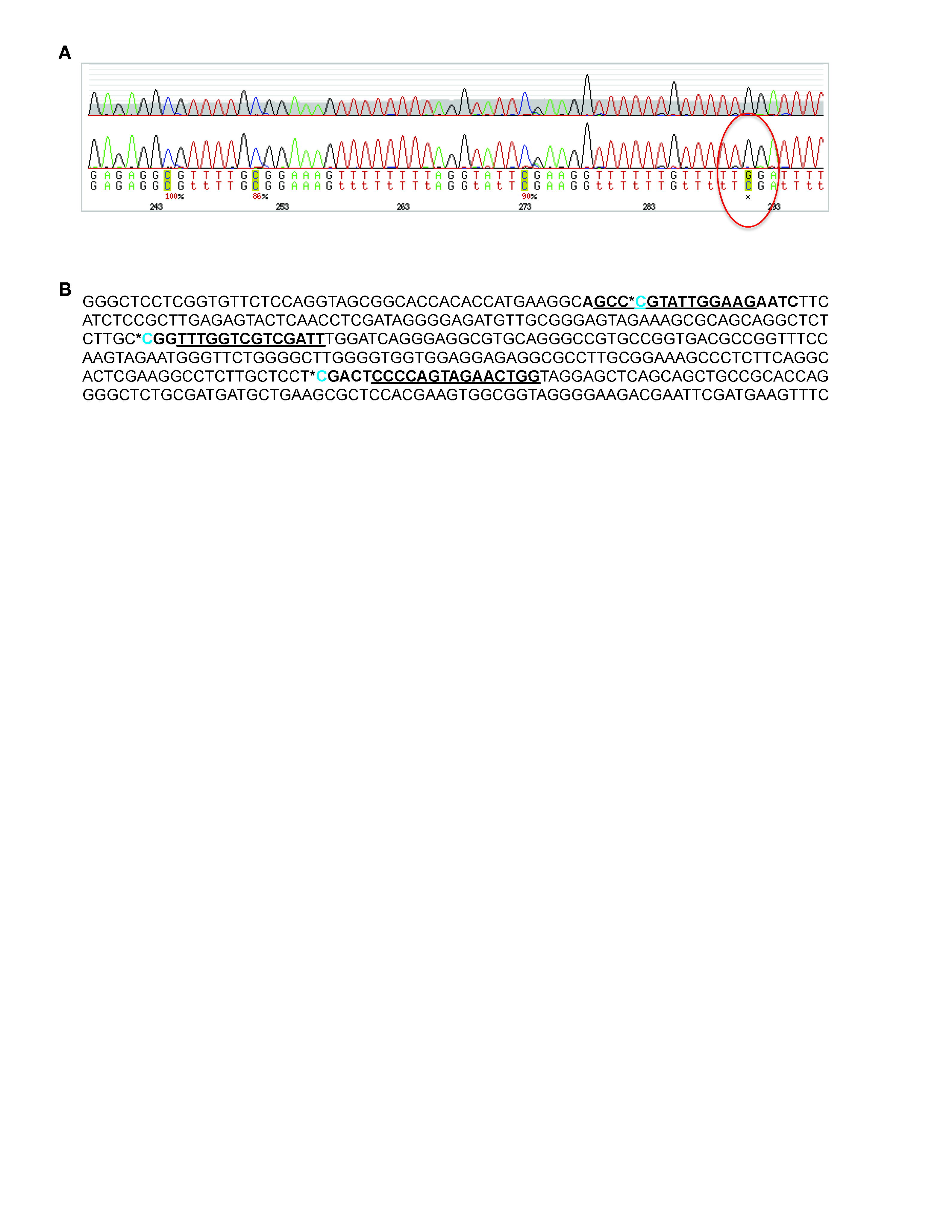

Supplement: S6 Fig — Transcription factor binding analysis revealed consensus sequences for the C/EBP (bold) and p300 (underlined) complex at all significantly decreased methylation sites indicated with an asterisk (*). The representative sequencing chromatogram depicts the cause for the lack of methylation data from obese subjects at the -732 site was due to a SNP (rs11623267). (TIFF) [file pone.0160057.s008.tiff]

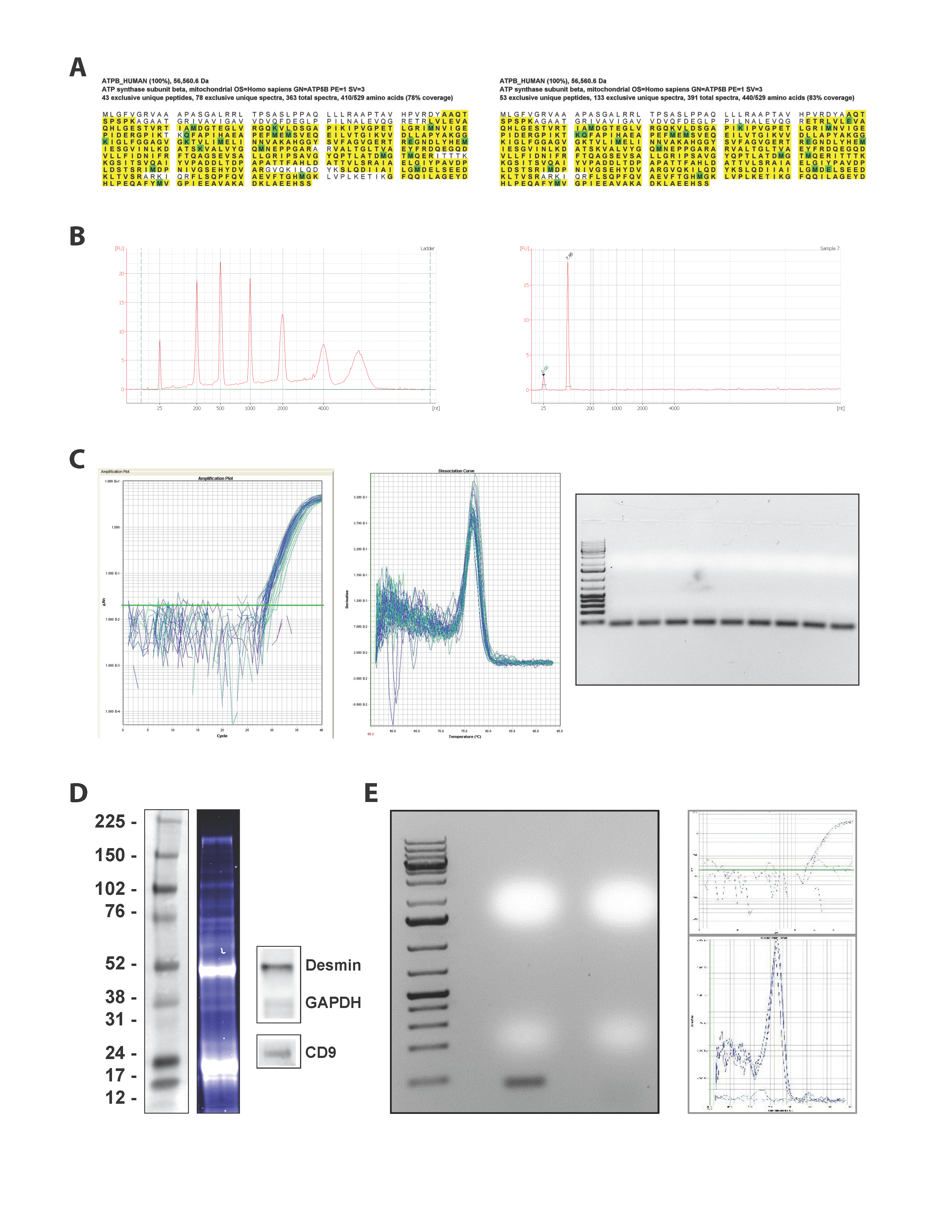

Supplement: S7 Fig — (A) LC-MS/MS verification of β-F1-ATPase protein in the ~50 kDa band precipitated by the β-F1-ATPase antibody. (B) Bioanalyzer results of RNA ladder (left) and total RNA extracted from exosomes (right) showing presence of small RNAs. (C) Amplification curve, melting curve, and DNA agarose gel for qRT-PCR analysis of hsa-miR-127-5p expression in serum exosomes. (D) Total proteins extracted and Western blot analysis of desmin (~50 kDa), GAPDH (~37 kDa), and CD-9 (~27 kDa) from serum exosomes. (E) DNA agarose gel, amplification curve, and melting curve for qRT-PCR analysis of hsa-miR-127-5p expression in exosomes extracted from myotube culture media. (TIFF) [file pone.0160057.s009.tiff]

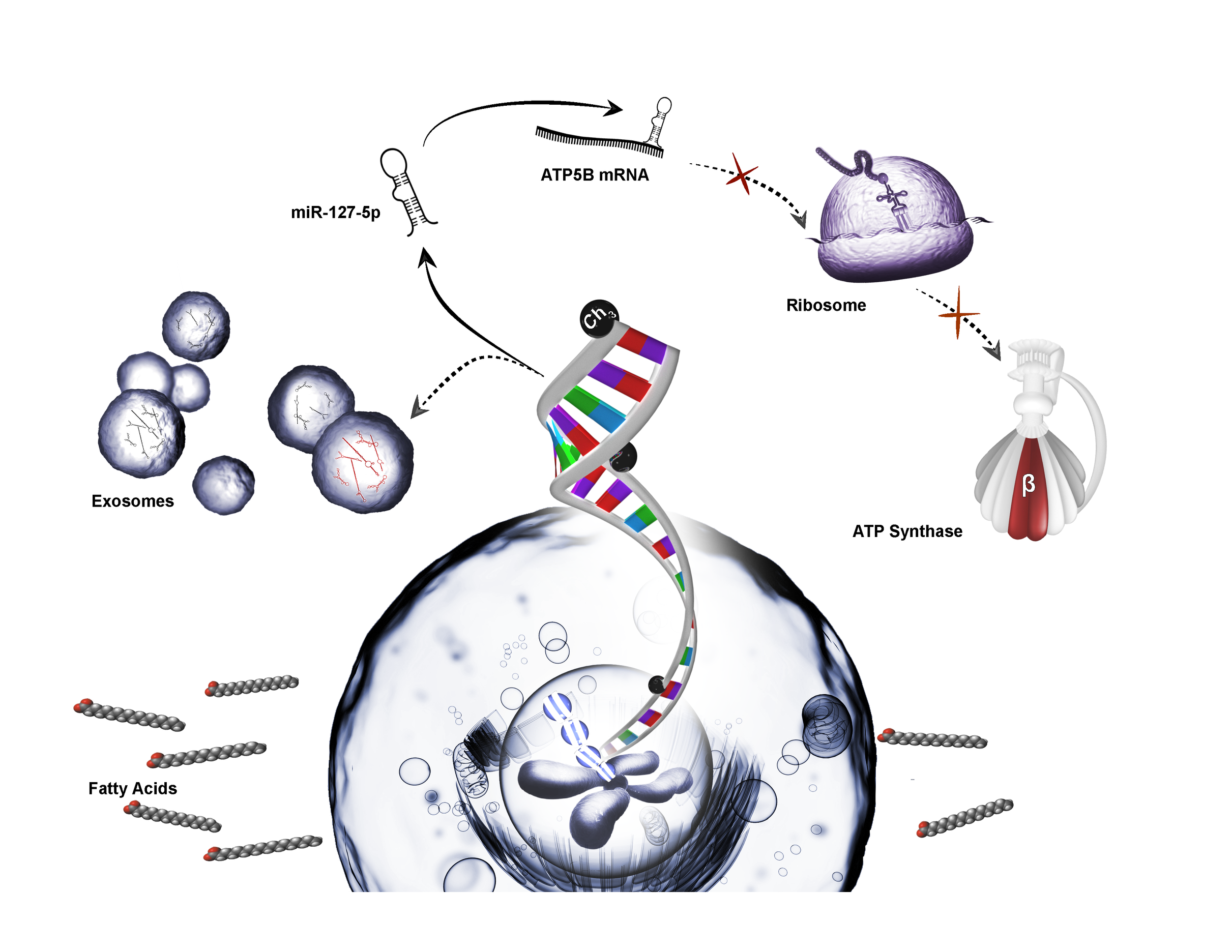

Supplement: S8 Fig — Genetic and epigenetic factors unmask transcription of miR-127-5p, which can then bind to the 3’ UTR of the ATP5B mRNA and block ribosomal translation machinery from accessing the transcript. (TIF) [file pone.0160057.s010.tif]

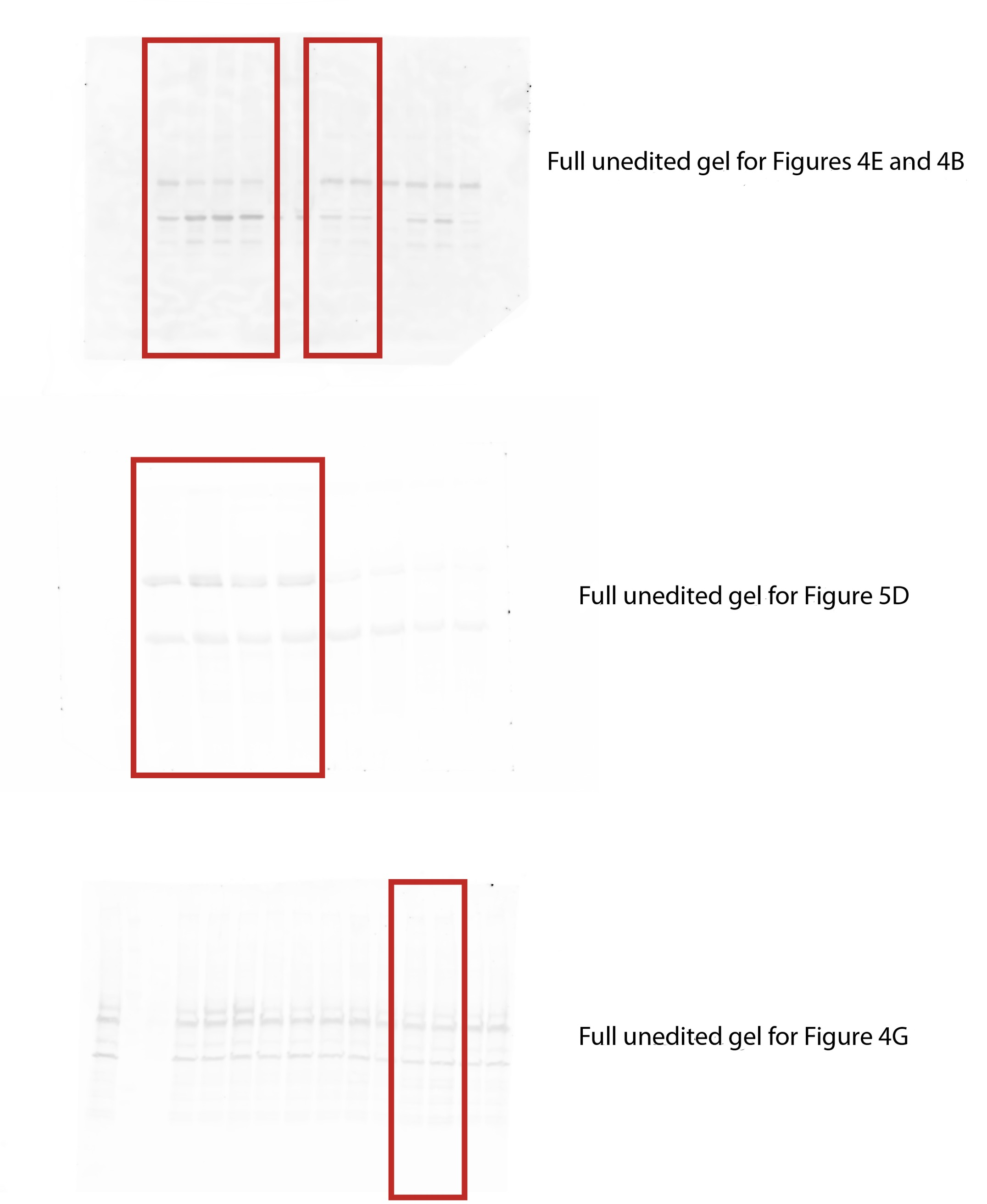

Supplement: S9 Fig — Original, uncropped, and unadjusted western blots for the data presented in the Figures in the Results Section. (TIF) [file pone.0160057.s011.tif]
